# Inflammation induced by snake venoms optimizes envenomation

**DOI:** 10.1113/JP290494

**Published:** 2026-06-08

**Authors:** Dirk F. van Helden, Neil Spratt, Peter J. Dosen, Sally A. McFadden, John Holdsworth, Daniel J Beard, Kirsten Coupland, Phillip Jobling, Todd A. Hardy, Derek R. Laver

**Affiliations:** ^1^ School of Biomedical Sciences and Pharmacy, College of Health, Medicine and Wellbeing University of Newcastle Newcastle New South Wales Australia; ^2^ Heart and Stroke Program, Hunter Medical Research Institute Newcastle New South Wales Australia; ^3^ Department of Neurology John Hunter Hospital, Hunter New England Local Health District Newcastle New South Wales Australia; ^4^ Department of Ophthalmology, Yong Loo Lin School of Medicine National University of Singapore Singapore; ^5^ School of Information and Physical Sciences, College of Engineering, Science and Environment University of Newcastle Newcastle New South Wales Australia; ^6^ Department of Neurology, Concord Hospital University of Sydney Sydney New South Wales Australia

**Keywords:** envenomation, inflammation, lymphatics, snakebite first aid, vasculature, venoms, venules, wound healing

## Abstract

**Abstract:**

Snake envenomation activates the immune system through permeability‐increasing factors that generate an acute vascular inflammatory response by opening large inflammation‐activated pores (IAPs) in the microvasculature. These events facilitate the exudation of plasma from blood into body tissues. However we show that IAPs also allow macromolecules, including venom toxins, to flood directly into the bloodstream, even against an outflow of plasma solutes. Such inflammation‐facilitated macromolecular absorption (IFMA) acts together with lymphatic absorption and has a physiological role in removing interstitial molecules, as evidenced by our dextran studies. IFMA will function in vascular absorption of interstitial molecules, potentially up to the radius of IAPs, which we determined to be 21 nm (95% confidence interval (CI) 18–24 nm). This absorption depends on factors, including the interstitial–vascular concentration gradient, the reflection coefficient of each molecule and microvascular pressure. Molecules absorbed will include snake venom toxins and various cellular breakdown products that arise during processes such as the inflammatory phase of wound healing. Most, if not all, venom toxins will be absorbed, as these typically have a hydrodynamic radius (*r*_0_) of 1–6 nm, which is well below that of IAPs. Notably once in the circulation venoms cause distributed inflammation, enhancing venom toxin movement from the bloodstream into the tissues. These mechanisms markedly increase the absorption of often lethal snake toxins and ensure their dissemination throughout body tissues, facilitating prey capture and adding to make snakebite extremely dangerous to humans. These findings provide mechanistic insight into current empirical snakebite first aid and present directions that may improve these procedures.

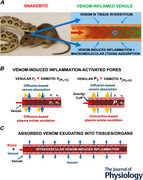

**Key points:**

- Snake venoms induce acute vascular inflammation, manifested by the opening of large pores termed inflammation‐activated pores (IAPs), which we experimentally measure to have a radius of 21 nm.
- These pores mediate well‐known exudation of plasma proteins but simultaneously provide a pathway for absorption of macromolecules, including venoms, a process that operates in parallel with the lymphatic system in clearing interstitial macromolecules.
- Although utilized in snakebite envenomation, such inflammation‐facilitated macromolecular absorption (IFMA) is likely to have important physiological or pathophysiological roles, candidates including clearance of cellular breakdown products during the inflammatory phase of wound healing.
- Once in the vasculature venoms induce distributed inflammation of the IAPs, facilitating the exudation of venom toxins into tissues.
- The findings together with a model based on our experimental data provide new directions for improving snakebite first aid and present experimental evidence for a mechanism that operates to clear interstitial macromolecules.

## Graphical abstract

**Abstract figure legend** Schematic illustrating mechanisms underlying direct vascular absorption and subsequent movement of venom toxins into the tissues consequent to snakebite. *A*, snakebite into prey deposits venom toxins into the tissue interstitium, with the venom then causing acute inflammation primarily in venules. *B*, venom toxins directly absorb into the bloodstream by diffusing through inflammation‐activated pores (IAPs). Direct vascular absorption is inhibited with increases in venular pressure (*P* **_V_**) (e.g. for bites to human lower limbs when standing or due to a pressure cuff) because of counteracting plasma outflow (see text for lymphatic contribution). *C*, venoms once in the bloodstream cause widespread inflammation and distributed opening of IAPs, markedly enhancing distributed venom toxin movement from the blood into the tissues. Images in *A* are generated by ChatGPT‐4o (Open AI, 2025), and those in *B* and *C* are partly created using BioRender (version 3.0; April 2026). Figure compiled and partly annotated using Microsoft PowerPoint. Abbreviations: π_v_ and π_i_, blood and interstitial colloid osmotic pressure, respectively; *P*_v_, intravenular pressure; *P*_i,_ interstitial pressure.

## Introduction

Venomous snakes have evolved venoms that optimally kill prey or predators. In the case of humans snakebite causes substantial loss of life and injury (Warrell, 2010). It is estimated there are at least 80,000‐138,000 deaths/year from snakebite globally with four times more physical disabilities (Gutierrez et al., 2017; WHO, 2021; Warrell & Williams, 2023) with another survey reporting 39,000‐79,000 snake bite‐related deaths/year (Collaborators, 2022). Although antivenom treatment remains fundamental, the rapidity of absorption of life‐threatening venom toxins means that many victims receive antivenoms too late, highlighting the need for reliable first‐aid procedures.

Snakebite venom absorption into the circulation occurs through direct entry into blood vessels and indirectly via lymphatics (Paniagua et al., 2012; Saul et al., 2011; Sutherland et al., 1979; van Helden et al., 2019; Vergara et al., 2016). Over the first few hours direct venom absorption into blood is greater than by the indirect lymphatic pathway, at least in vasculature near heart level where hydrostatic forces are minimal, as demonstrated in studies on anaesthetized sheep (Paniagua et al., 2012) and rats (van Helden et al., 2019). This vascular condition will be the case for most snake prey (e.g. reptiles, amphibia, rodents) and likely the same for human victims who lie down after being bitten. Surprisingly the basis for direct absorption of venom toxins into the vasculature remains poorly understood.

Snake venoms generally cause inflammation, with consequences such as increased vascular leakage of plasma, causing localized oedema and pain (Brain et al., 1977; Feldberg & Kellaway, 1938; Gutierrez et al., 1980; Landucci et al., 1998; Maia‐Marques et al., 2021; Miller & Tu, 1989; Rao et al., 2024; Teixeira et al., 2003). Acute vascular inflammation is caused by permeability‐increasing factors (PIFs), including histamine, serotonin and vascular endothelial growth factor (VEGF) (Dvorak et al., 1979; Majno & Palade, 1961; Nagy et al., 2012; Pappenheimer & Soto‐Rivera, 1948; Renkin et al., 1974; Rippe & Haraldsson, 1994; Roberts & Palade, 1995), which induce microvascular leakage through opening of large inflammation‐activated pores (IAPs), primarily in venules (Ashina et al., 2015; Egawa et al., 2013; Galli et al., 2013; Levick & Michel, 2010; Majno & Palade, 1961; Nagy et al., 2012; Rippe & Haraldsson, 1994; Theoharides et al., 2023). IAPs are generated by endogenous mechanisms activated by these PIFs. A factor that is stimulated by PIFS, such as histamine, is endothelial nitric oxide synthase (eNOS), causing enhanced production of nitric oxide (NO) (Ashina et al., 2015; Bucci et al., 2005; Mayhan, 1994). NO leads to relaxation of arterioles, resulting in changes including increased microvascular pressure, blood flow, wall distension, vascular permeability and systemic changes in vascular endothelial cadherin (Mayhan, 1992, 1994; Ashina et al., 2015; Corada et al., 2001; Feletou et al., 1996; Nan et al., 2023). Together these and other factors combine to open IAPs.

We report here that acute vascular inflammation caused by opening of IAPs, as induced by snake venoms or other inflammatory factors, substantially increases the absorption of macromolecules. The dominance of this mechanism in rapidly clearing tissue macromolecules, including venoms that are present at higher levels in the interstitium than that in the blood, makes it of considerable physiological and pathophysiological interest. Furthermore once in the circulation venoms induce systemic inflammation, facilitating macromolecular exudation widely into body tissues. These attributes have presumably evolved in snakes to markedly enhance their envenomation.

## Methods

### Ethical approval

The experimental protocols were approved by University of Newcastle Animal Care and Ethics Committee (approvals A‐2009‐153 A‐2021‐123). They comply with the criteria of the Australian Code of Practice for the Care and Use of Animals for Scientific Purposes as set out by the National Health and Medical Research Council of Australia (2004). This NHMRC Code supports the principles that are consistent with ARRIVE guidelines 2.0.

### Experimental model

Studies were conducted using male and female Wistar rats (85% male; weight range 160–400 g, with 90% in range 250–380 g of ∼age range 10–20 weeks; *N* = 367). All animals were bred and maintained in the University of Newcastle (Australia) Bioresources Facility, a modern, high‐standard animal research environment with strong infection control (SPF conditions) They were housed in individual ventilated cages with environmental enrichment, paper‐based crinkle bedding and 2–3 (typically 2) males or females per cage (depending on weight). Animals were exposed to a 12 h light/dark cycle with *ad libitum* access to food and water. Temperature and humidity ranged between 20°C and 22°C and 40% and 50%, respectively. The number of animals used was minimized where possible by performing experiments that included making simultaneous measurements from different skin regions of the same rat (see below).

All experiments were conducted on these rats during deep non‐recovery anaesthesia. Atropine (0.2 mg/kg) was injected subcutaneously in the dorsal cervical region prior to anaesthesia to reduce salivary and airway secretions and to mitigate vagal reflexes. Anaesthesia was achieved by intraperitoneal (i.p) injection of urethane in two stages: the first dose was 1 g/kg followed by half of this dose injected 10–20 min later. Additional top‐up doses were only occasionally required. Urethane was used as it induces surgical anaesthesia without inhibiting peripheral neurotransmission (Maggi & Meli, 1986). Experiments were generally conducted for ≤ 2 h, although a few (∼5%) were continued for up to 4 h. Rats were killed by intravenous (IV) or i.p injection of an overdose of urethane (1–2 g/kg), with animals monitored to ensure that respiration had ceased, the heart had stopped beating and the rat had no reflexes. Some of the rats died from the venom itself, but always while fully anaesthetized. Body temperature was maintained at 35°C–36°C throughout. Some experiments involved cannulation of the carotid artery and/or jugular vein for measurement of heart rate, blood collection or IV injection. In such cases heparin sulphate (10 units/ml) was included in the cannula for the longer experiments to prevent clotting during blood collection. Video recording was used in some experiments to monitor rat breathing rate.

### Venoms and drugs

Snake venoms used included those from Eastern Brown snake – *Pseudonaja textilis* (PtV), death adder – *Acanthophis antarcticus* (AaV), Russell's viper – *Daboia russelii* (DrV), Stephen's banded snake – *Hoplocephalus stephensii* (HsV) and Eastern mainland tiger snake – *Notechis scutatus* (NsV). The Australian Reptile Park (Somersby NSW Australia) and Venom Supplies (Adelaide, South Australia) provided these venoms in lyophilized form. They were made up to stock concentration in phosphate‐buffered saline (PBS). Fluorescent agents used were Evans blue (Sigma‐Aldrich) and fluorescein isothiocyanate (FITC)‐dextrans (FDs; Sigma‐Aldrich) of molecular sizes 10, 20, 40, 70 and 250 kDa (i.e. FD10–FD250). Other pharmacological agents were cetirizine dihydrochloride (CD; H1 histamine antagonist), methysergide maleate (MM; serotonin antagonist) and sodium cromoglycate (Cr; mast cell stabilizer), all obtained from Sigma‐Aldrich. We also used a commercially available NO donor ointment (glyceryl trinitrate‐GTNO, 0.2% wt/wt; Care Pharmaceuticals) and an antivenom (AV; Commonwealth Serum Laboratories (CSL) brown snake antivenom).

### FITC dextran calibration

*In vitro* calibration measurements were made using different‐sized FITC dextrans for a range of concentrations. Samples were dissolved in water and spotted at 20 µl onto a glass slide with fluorescence measured using a high‐sensitivity camera (Andor iXon EMCCD, Andor technology, Belfast, Northern Ireland) illuminated for FITC fluorescence (excitation: lens‐dispersed 488 nm laser; emission: 512 nm high‐pass filter). Resultant fluorescence was captured using Imaging Workbench (INDEC Biosystems, CA, USA) on a PC computer. Imaging was repeated with the slide turned 180 degrees with the intensity of the corresponding spots averaged. Fluorescence intensity of the spots was measured using Image J (NIH, USA), and the data were collated in Microsoft Excel. FD10, FD20, FD40, FD70 and FD250 exhibited similar levels of fluorescence at any set FD concentration ([FD]; *N* = 3 for each dextran). This was upheld for a range of FD concentrations (i.e. 0.15, 0.5, 1.5 and 5 µg/ml). The average fluorescence of these data at each concentration increased linearly with [FD], with the measured relative fluorescence of the combined data plotted against [FD] exhibiting a slope of 6.0 fluorescent units/(µg/ml) and intercept of 1.1 fluorescent units (R^2^ = 0.93). This relationship allows the estimation of the [FD] absorbed in blood based on the level of FD fluorescence exhibited in serum.

### Procedures

- *FITC‐labelled dextran (FD) absorption measured from blood serum samples ± pressure cuff*.

Dextran absorption was measured from blood samples consequent to subcutaneous injection into the rat hind paw of a bolus (usually ∼50 µl) of FD (10–250) ± venom ± pharmacological agent(s) or intravenous (IV) injection of FD10 (1 mg in a 50 µl PBS bolus) into the cannulated jugular vein. Some experiments also included the use of a pressure cuff on the same hind limb as the injection, applied within 1–2 min after hind paw injection. Blood (∼100 µl) was sampled before injection (control sample) and at selected intervals after injection and then stored on ice in labelled centrifuge tubes for 0.5–2 h depending on the collection time. Serum was prepared by removing the samples from the ice, allowing the blood to clot in the samples over the next 15–30 min and then spinning down these samples at a speed of ∼3500 rpm for 10 min using a benchtop centrifuge. A 20 µl aliquot of serum was then sampled from each tube and spotted onto a glass slide in two or three rows. The serum spots were imaged with a high‐sensitivity camera (Andor iXon EMCCD) illuminated for FITC fluorescence (excitation: lens‐dispersed 488 nm laser; emission: 512 nm high‐pass filter). Data were captured using Imaging Workbench (INDEC Biosystems) on a PC computer. Imaging was repeated with the slide turned 180 degrees, with the intensity of the corresponding spots averaged. Fluorescence intensity of the spots was measured using Image J (NIH, USA), with data collated in Microsoft Excel. The final data were baseline corrected by subtracting the background fluorescence of the control serum sample. Data are presented as fluorescence values relative to the mean fluorescence level of the plateau response to hind paw injection of FD10 + PtV.

(b) *Venom FD absorption and consequent exudation into the interstitium of the ear pinna*.

This experimental procedure involved rat hind paw injection of FD ± venom ± pharmacological agent(s) ± leg‐applied GTNO ointment (LGTN). FITC‐labelled dextran fluorescence (excitation: lens‐dispersed 488 nm laser; emission: 512 nm high‐pass filter) was measured from the glabrous region of the ear (i.e. pinna) using an Andor iXon EMCCD camera, with images taken every 10 s. The same ear pinna imaging procedure was used following IV FD injection. Data were captured using Imaging Workbench (INDEC Biosystems) on a PC computer and analysed using Image J (NIH) and Microsoft Excel. Imaging data were baseline corrected by subtracting the fluorescence typically averaged at times 1.5–2.5 min after commencement of recording before the onset of a measurable response (NB: data obtained over the first minute were set to zero due to artifacts associated with positioning). Resultant fluorescence data were divided by the background fluorescence and plotted in normalized form relative to the plateau response to hind paw injection of FD10 + PtV. Blood serum FD levels were measured as in (a), using blood samples collected  < 5 min before and at the end (∼120 min) of the imaging.

(c) *Inflammation induced vascular exudation by fluorescently imaging Evans blue‐labelled albumin (EB‐albumin) in posterior rat hind skin*.

This study utilized an approach developed to study vascular exudation of plasma and its solutes by measuring the exudation of albumin in response to histamine in rodent skin (Miles & Miles, 1952). Albumin in the bloodstream was labelled with Evans blue dye (EB) by IV injection of 20 mg/kg EB at least 5 min before the commencement of experimental recording, with this binding to > 99% of the albumin (Freedman & Johnson, 1969). Local boluses, typically ∼50 µl of venom ± pharmacological agents (in a few cases 5 µl volumes used), were then subcutaneously injected at several non‐overlapping sites into rat posterior hind skin (i.e. posterior dorsal dermis) previously depilated by a commercial hair remover (Nair, Church & Dwight Co. Inc., Ewing, New Jersey, USA); the combined SC injections administered in < 2 min. The venoms and doses used were AaV, HsV, NsV, PtV, all at 0.05–0.15 mg/kg, and DrV at 0.1–0.2 mg/kg. Test agents used were CD (0.8 µg), Cr (2.5 µg) and MM (8 µg). These agents were not animal weight corrected given their local actions. Some experiments were made with antivenom (AV; 4.5 units/kg) ± PtV (0.05–0.1 mg/kg) ± pharmacological agent(s). Exudation was measured simultaneously at injected sites using an Andor iXon EMCCD camera to image EB‐labelled albumin fluorescence (excitation: lens‐dispersed 488 nm laser; emission: 630 nm high‐pass filter; power: ∼10 mW). This procedure is based on a known property of EB, which exhibits fluorescence peaking near 680 nm when excited by 488 or 540 nm light (Hed et al., 1983). Data were captured at 10 s intervals using Imaging Workbench (INDEC Biosystems) on a PC computer, with recordings commenced within 2 min immediately after completion of injections and continued for up to 2 h. Fluorescence intensity was analysed using Image J (NIH) and Microsoft Excel. Data were baseline corrected by subtracting the fluorescence averaged over ∼1 min starting 1–2 min after commencement of video recording before the onset of a measurable response. Data were plotted in normalized form (Prism).

### Statistical analysis

Excel (Microsoft) or Graphpad10 Prism for MacOS (Dotmatics, USA) was used for statistical analysis. The sample size (*N*) refers to the number of animals with measurements for any specific parameter made from different animals. Comparisons for two datasets were made using either *t* tests or multiple *t* tests (two‐sided, paired or unpaired, unequal variance) for data that passed normality tests (Kolmogorov–Smirnov test) or by non‐parametric Mann–Whitney tests (two‐sided) where ranks were compared. Statistical comparison for three or more datasets used two‐way ANOVA, with multiple comparisons made using Tukey's multiple comparisons test. Data were considered significant for *P‐*values < 0.05. Data are presented as means ± SEM, with *N* specified. Data were plotted using GraphPad Prism.

The effects were generally very large as indicated by preliminary experiments. Experiments were usually replicated with five or more measurements repeated on different animals except in a few cases where a group showed very little variance and formed a component of a larger dataset. Data were excluded in ∼5% of cases with the criteria that the specific data differed by greater than the mean + 2 × SD, instances that could be caused by damaging a blood vessel.

### Modelling

Mathematical approaches were used to model the diffusion of macromolecules through IAPs (see Appendix). The resultant equations provided insight into inward movement of macromolecules dominant in the interstitium against outward movement of plasma proteins as a function of venular pressure. Simulations of the outcomes that these equations predicted were made using Excel, resulting in the theoretical plots presented in the Results section.

Least‐squares fitting using the MATLAB fit function lsqcurvefit was employed to determine the radius of the IAP and the colloid osmotic pressure. The 95% confidence intervals (CIs) were determined using MATLAB function nlparci, which computes CIs using a linearization (Wald‐type) method based on the Jacobian at the solution. It assumes that the non‐linear model is approximately linear near the best‐fit parameters and propagates the residual variance through the Jacobian to estimate parameter uncertainty. Fitting was performed using data in log‐linear form.

## Results

### Inflammation facilitated macromolecular absorption

**Figure 1:**
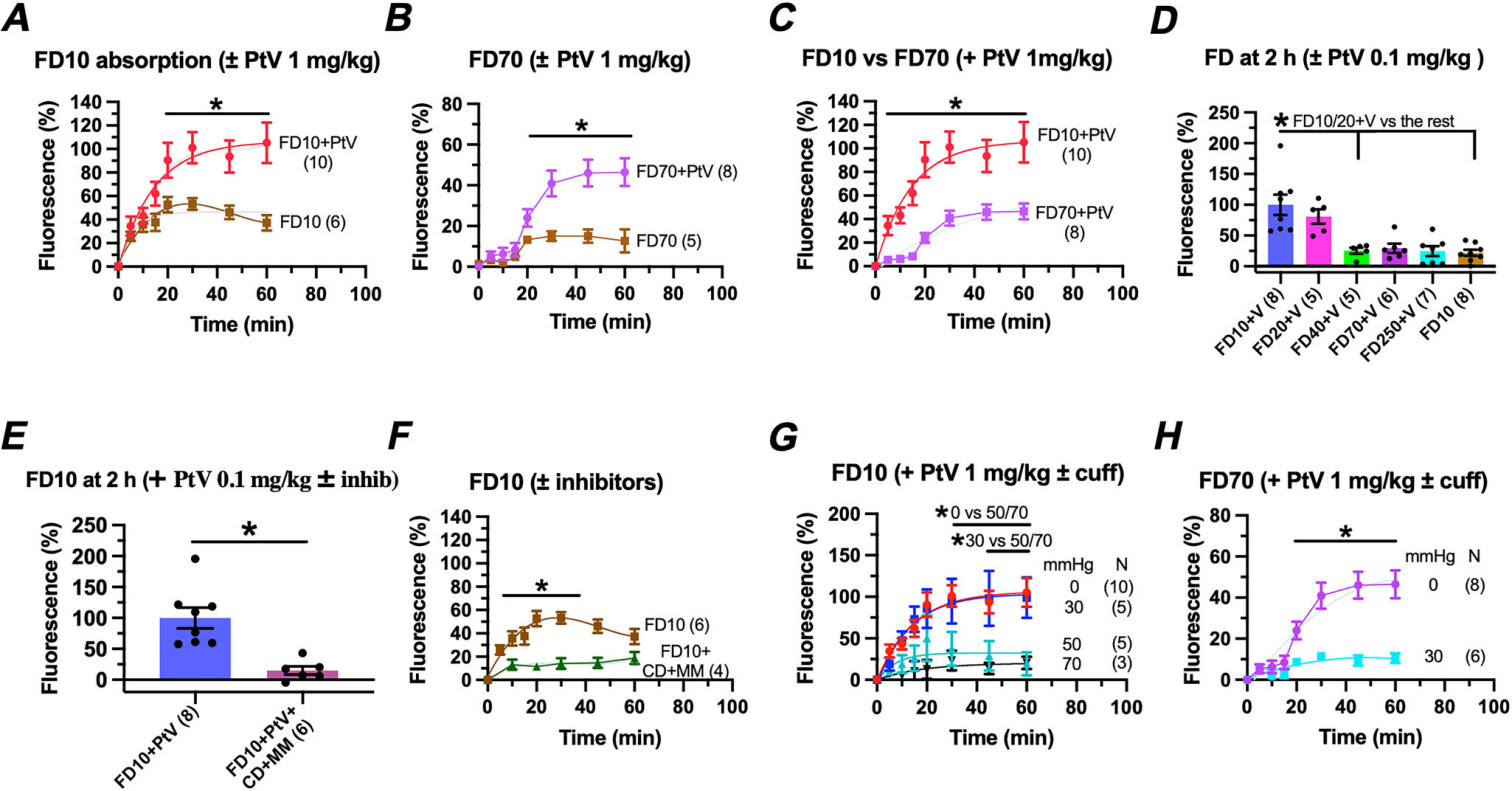
Venom‐induced dextran absorption *A*, serum fluorescein isothiocyanate (FITC) fluorescence in response to hind paw co‐injection into anaesthetized rats of an elapid venom (PtV at 1 mg/kg) together with a FITC‐labelled 10 kDa dextran (FD10 in ∼50 µl phosphate‐buffered saline (PBS) bolus) compared to that in response to injection of FD10 without venom (same bolus size). Data were normalized to the 30–60 min plateau of the FD10 + PtV response. *B*, response to FD70 + PtV (1 mg/kg) *versus* FD70 using the same methodology as for *A*, with normalization to the plateau of the FD10 + PtV response of *A*. *C*, comparison of the FD10 +PtV response of *A* to the FD70 + PtV response of *B*. *D*, comparison of serum fluorescence to hind paw co‐injection of FDs of molecular weights 10, 20, 40, 70, 250 kDa + PtV (0.1 mg/kg) and FD10 without PtV measured 2 h after hind paw injection, with data normalized to the mean FD10 + PtV response from this dataset. *E*, comparison of the FD10 + PtV data of *D* compared to the same co‐injection but with further addition of the antihistamine CD (0.8 µg) together with the serotonin antagonist MM (8 µg). *F*, the response to FD10 (without venom) of *A* was inhibited by hind paw co‐injection of FD10 together with cetirizine dihydrochloride (CD) and methysergide maleate (MM) (as for E). *G*, the effects of a pressure cuff on serum FD10 levels to hind paw injected FD10 +PtV (1 mg/kg) for cuff pressures of 30, 50 and 70 mmHg compared to without a cuff (from *A*). *H*, the effects of a pressure cuff at 30 mmHg on serum FD70 levels compared to without a cuff (from *B*) using the same protocol as for *G*. Data in *G* and *H* were normalized to the mean 30–60 min plateau of the 0 mmHg response to FD10 + PtV. FDs were all applied at 1.5 mg/kg. Artifacts during first 0–1 min removed. Statistics: means ± SEM (*N*‐number of animals); two datasets: *t* test or Mann–Whitney test (MW); > 2 data sets – two‐way ANOVA with Tukey's multiple comparisons test (ANOVA); significance: *P*‐values denoted by asterisks ranged from *A*, 0.0312–0.00159 (MW); *B*, 0.0295–0.00621 (MW); *C*, 0.0067– < 0.001 (MW); *D*, 0.0424–0.002 (ANOVA); *E*, 0.007 (MW); *F*, 0.0381–0.0095 (MW); *G*, 0.014–0.0069 (ANOVA); *H*, 0.0079–0.0013 (MW).

In non‐recovery urethane anaesthetized rats hind paw co‐injection of a 10 kDa FITC‐labelled dextran (FD10) together with venom from the common brown snake (*P. textilis*; PtV) significantly enhanced FD10 fluorescence in the serum which plateaued at ∼30 min (Fig. 1*A*). The 30–60 min plateau amplitude of fluorescence for the FD10+PtV response before normalization to this plateau level was 10.5 fluorescence units, which based on our calibration corresponded to an FD10 concentration in the serum of 0.16 µg/ml (see Methods). Venom (FD10 + PtV at 1 mg/kg) increased the level of the response by 50% compared to the control (FD10 in PBS) injection (0–60 min area under the curve (AUC) comparison; Fig. 1*A*). Absorption following hind paw co‐injection of this same dose of PtV together with 70 kDa FITC‐labelled dextran (FD70) was increased by 60% compared to FD70 without PtV (Fig. 1*B*; 0–60 min AUC comparison). FD70 + PtV absorption was 60% lower than that for FD10 + PtV (Fig. 1*C*). This difference in absorption was investigated for a wider range of fluorescent dextran sizes (i.e. FD10, 20, 40, 70, 250 kDa) measured in a different series of experiments 120 min after hind paw injection of FD10 without venom or injection of FD(10–250) + PtV at a lower dose of 0.1 mg/kg (Fig. 1*D*).

This same experimental protocol was used to investigate lymphatic contribution to absorption of dextran FD10 in response to PtV. We investigated the effect of inhibiting lymphatic function by application of leg GTNO (LGTN) (Saul et al., 2011). The mean fluorescence values normalized to control and measured ∼2 h after injection consequent to hind paw injection of PtV (0.1 mg/kg) + FD10 (1.5 mg/kg) + LGTN being 81 ± 27% (7) compared to 100 ± 17% (8) for the PtV + FD10 control. This difference was not significant (*P = *0.562, *t* test).

A role for acute vascular inflammation in response to hind paw injection of FD10 + PtV (0.1 mg/kg) as measured 120 min after injection was indicated by the finding that co‐injection of the antihistamine (CD) together with the serotonin antagonist (MM), namely FD10 + PtV + CD + MM, caused large inhibition (Fig. 1*E*). Unexpectedly hind paw injection of FD10 without venom caused transient inflammation, as indicated by the fact that the addition of the antihistamine and the serotonin antagonist to FD10 injection (i.e. FD10 + CD + MM) caused 70% inhibition (0–60 min AUC comparison; Fig. 1*F*). The inflammatory response to hind paw injection of FD10 in PBS without venom lasted ∼1 h after which time the effects dissipated (Fig. 1*F*).

Taken together these data indicate that venom co‐injection facilitates dextran macromolecular absorption by generating acute vascular inflammation primarily through the venom but also through a transient inflammation associated with the hind paw injection *per se*. Using the FD10 +CD+MM record (Fig. 1*F*) as a baseline and comparing this to the inflammatory response to FD10 + PtV of Fig 1*A* indicate that inflammation increased FD10 absorption by 610% (0–60 min AUC comparison from mean records). The findings also demonstrate that smaller macromolecules (i.e. FD(10, 20) + PtV are more readily absorbed into the bloodstream compared to FD(40, 70, 250) + PtV or FD10 hind paw injected without venom.

### Inhibition of absorption by a venous pressure cuff

A first‐aid method recommended for elapid snakebite in Australia involves pressure bandaging with immobilization (PBI), an empirical technique shown in animal experiments to inhibit the absorption of various snake venoms (Meggs et al., 2010; Sutherland et al., 1979; Sutherland et al., 1981; van Helden et al., 2019). We studied the effectiveness of this procedure on inhibiting absorption for different‐sized macromolecules by investigating the relative effect of a leg‐applied venous pressure cuff in inhibiting absorption of 10 and 70 kDa dextrans co‐injected with PtV (1 mg/kg) in anaesthetized non‐recovery rats. The cuff was applied to the same hind limb in which the FD + PtV (1 mg/kg) was hind paw co‐injected. A 50 or 70 mmHg cuff pressure (*P*_cuff_) substantially inhibited FD10 absorption, whereas a *P*_cuff_ of 30 mmHg did not (Fig. 1*G*). In contrast absorption of FD70 was largely inhibited with a *P*_cuff_ of 30 mmHg (Fig. 1*H*). These data indicate that absorption is more readily inhibited with cuff‐increased venous pressure for larger macromolecules than for smaller ones. Furthermore although the pressure cuff is well known to inhibit lymphatic function (Howarth et al., 1994), it also simultaneously inhibits direct vascular uptake, which was the dominant pathway under the conditions of our experiments.

### Inflammation facilitated macromolecular exudation from the bloodstream to body tissues

**Figure 2:**
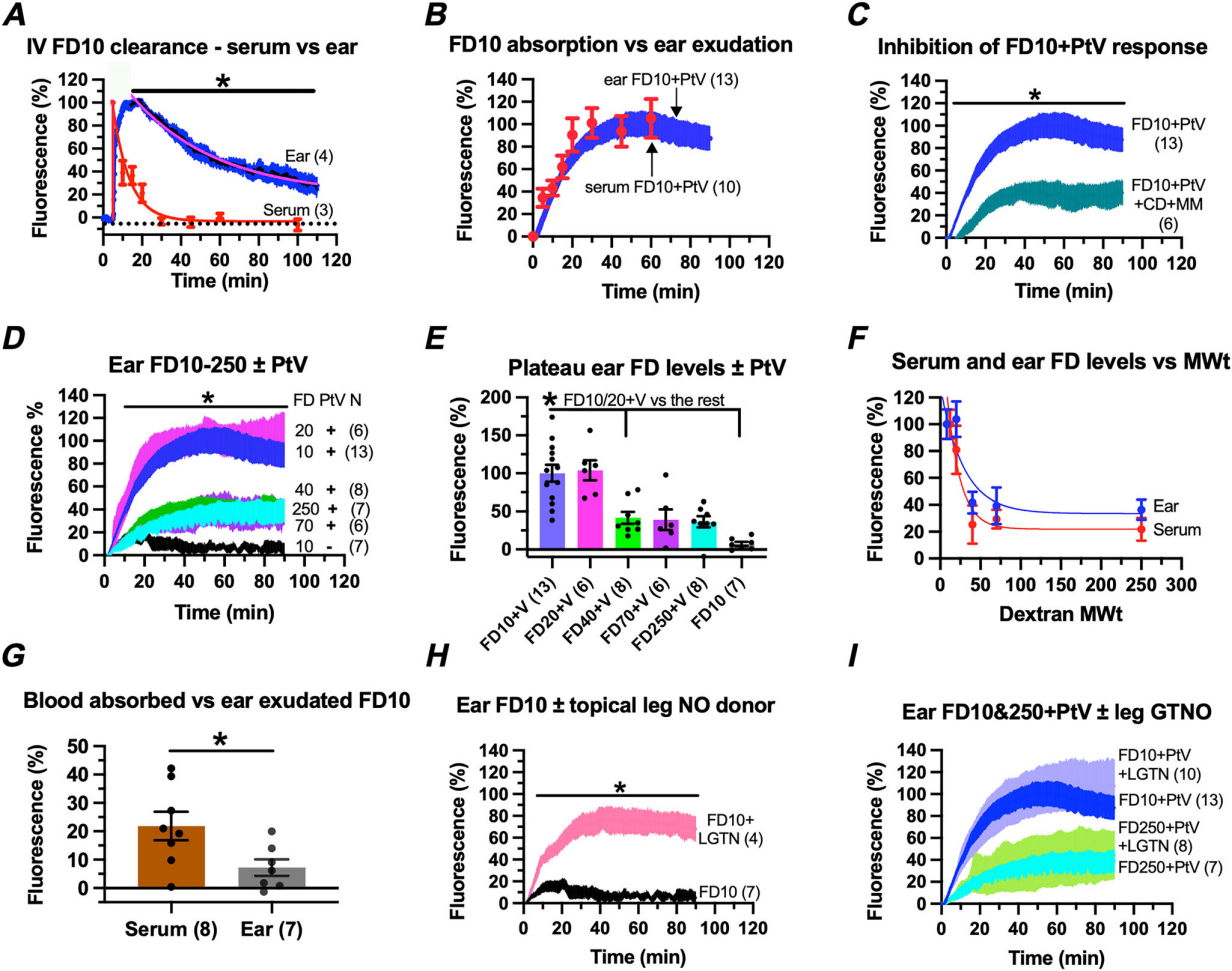
Dextran exudation into the ear interstitium *A*, serum *versus* ear FD10 fluorescence measured in separate experiments consequent to injecting 0.1 mg of FD10 IV in a 1 ml bolus, with records normalized to the peak respective data (mean ± SEM; exponential fits – Prism). *B*, normalized ear (blue) and serum (red; from Fig. 1*A*) FD10 fluorescence levels consequent to hind paw injection of FD10 + PtV (0.1 mg/kg ear; 1 mg/kg serum). *C*, ear FD + PtV fluorescence (duplicated from *B*) compared to the response for the same injection together with antihistamine CD (0.8 µg) + serotonin antagonist methysergide maleate (MM) (8 µg). *D*, comparison of the fluorescence response to hind paw injection of FDs ± PtV (0.1 mg/kg) of different molecular weights (range 10 – 250 kDa) normalized to the 50–60 min plateau FD10 + PtV response duplicated from *B*. *E*, comparison of the 50–60 min FD ear plateau fluorescence for the different‐sized dextrans measured from *D*. *F*, the 50–60 min plateau FD + PtV ear fluorescence from *E* compared to the serum fluorescence from Fig. 1*D*, both using the same injection protocol and normalized to their respective peak FD10 + PtV values. The curves are exponentials of best fit (Prism). *G*, relative FD10 serum fluorescence (from Fig. 1*D*) *versus* ear fluorescence (from Fig. 2*E*) in response to hind paw injection of FD10 (without venom) for both cases. *H*, ear fluorescence response to hind paw injection of FD10 ± leg applied nitric oxide donor LGTN (records normalized to the peak FD10 +PtV fluorescence response in D). *I*, normalized ear fluorescence responses to hind paw injection of FD10 + PtV (from B) or FD250 + PtV (0.1 mg/kg), both ± LGTN. FDs were all applied at 1.5 mg/kg. Artifacts during the first 0–1 min were removed. Statistics – see Fig. 1 legends. *P‐*value*s* ranged as follows: *A*, 0.027– < 0.001 (*t* test), *B*, 0.975–0.376 (not significant ‐ NS, Mann–Whitney test (MW)); *C*, 0.036–0.001 (MW); *D*, 0.045–0.004 (ANOVA); *E*, 0.034–0.001 (ANOVA); *F*, 0.535–0.214 (NS, *t* test); *G*, 0.040 (MW); *H*, (0.042–0.006 (MW); *I*, FD10: 0.997–0.879 (NS, MW); FD250 0.967–0.536 (NS, MW).

To rapidly immobilize prey snake venom toxins need to both enter the vasculature and then readily exudate into the tissues. The ear pinna proved a reasonably representative site for imaging the tissue interstitium, as ear clearance of FD10 consequent to IV injection was some fivefold slower (clearance half‐time (*T*_0.5_) = 31 min; 95% CI 28–34 min) than clearance from the blood (*T*_0.5 = _6.0 min; 95% CI 4.1–8.6 min; Fig. 2*A*). This suggests that pinna imaging will primarily reflect tissue dextran levels and not those in the blood. Another means of testing this is by comparing the tracer (i.e. FD10) in the blood and ear pinna using a two‐compartment model, one compartment representing the vasculature and the other representing the tissue. The time course of tracer in the blood consequent to IV injection had a rapid initial exponential rise with a time constant of 30 s and a decay time constant of 6 min, as derived from fits to the data in Fig 2*A* (red trace). Diffusion into the tissue was calculated from the concentration difference between blood and tissue and the ratio *V*_r_ of the tissue volume/vascular volume. The equations were integrated using Euler's method, and the total tracer (blood + tissue) was compared to the data. The data were well fit with *V*_r_ of 5, a value that corresponds with direct measurement of *V*_r_ of the rabbit ear, where a *V*_r_ of 4 was reported (Bent‐Hansen & Svendsen, 1991). A *V*_r_ of 5 indicates that approximately 80% of the fluorescent signal will arise from the ear pinna tissue, with the rest originating from blood.

Therefore the movement from blood to the tissues of different‐sized FDs was investigated by imaging the pinna consequent to subcutaneous injection of venom. The venom‐facilitated increase in FD10 ear tissue fluorescence consequent to hind paw co‐injection of FD10 + PtV (0.1 mg/kg) (Fig. 2*B*, blue record) paralleled that for FD10 absorption into the blood, as measured from serum for hind paw injection of FD10+PtV (1 mg/kg; serum data copied from Fig. 1*A*). This indicates that under venom‐inflamed conditions macromolecular exudation from the blood into the ear pinna interstitium is not rate limiting; therefore the kinetics of this response reflects absorption. Co‐injection of the PIF antihistamine CD together with serotonin antagonist MM (i.e. CD + MM) significantly decreased the ear fluorescence response to hind paw injection of FD10 + PtV (Fig. 2*C*). Fluorescence measurements of FD exudation into the interstitium of the ear pinna were compared for dextrans ranging in molecular weight. The ear fluorescence responses to hind paw co‐injection of dextrans FD(10–250) + PtV (0.1 mg/kg) and FD10 without venom and the corresponding 50–60 min plateau fluorescence levels are shown in Fig. 2*D* and *E*. The FD + PtV data normalized to the FD10 + PtV peak ear fluorescence response were not significantly different from the profile of blood‐absorbed FD levels (Fig. 2*F*), the latter measured in the same animals shortly after cessation of imaging (i.e. ∼120 min after injection), as previously presented (Fig. 1*D*).

A key difference between blood‐absorbed FD10 and resultant levels of exudated FD10 in the interstitium of the ear pinna consequent to hind paw injection of FD10 was that FD10 when injected without venom caused a smaller increase in relative ear fluorescence compared to that absorbed in the blood (Fig. 2*G*). This indicates that FD10 in the absence of venom does not itself cause inflammation once in the blood stream, a condition in which exudation from the blood to the ear becomes rate limiting compared to absorption. In contrast the enhancement of dextran exudation into the ear by venom indicates that absorbed venom initiates acute vascular inflammation within distributed vascular beds, leading to the opening of IAPs and subsequent exudation of macromolecules.

Venom‐independent evidence of the need for intravascular inflammation for enhanced macromolecular exudation into tissues was provided by the finding that topical hind limb application of NO donor ointment (LGTN) markedly increased exudation of hind paw‐injected FD10 from the bloodstream into the ear interstitium (Fig. 2*H*). This indicates that exogenous application of NO as donated by topical LGTN, which readily permeates into tissues, including lymphatic vessels (Saul et al., 2011), enters the vasculature to cause acute vascular inflammation. That NO causes such inflammation is consistent with reports that inhibitors of eNOS markedly diminish the acute vascular inflammation caused by PIFs such as histamine (Ashina et al., 2015; Bucci et al., 2005; Mayhan, 1994). Notably venom‐induced vascular inflammation caused by hind paw injection of FD + PtV and measured by the ear fluorescence response was not further enhanced by exogenous NO, as application of LGTN did not significantly alter venom‐facilitated FD10 or FD250 exudation from the bloodstream into the ear (Fig. 2*I*). This indicates that once acute vascular inflammation has been initiated by venom then exogenously enhancing NO has no further effect. Together these data indicate that inflammation is fundamental for exudation of macromolecules, including venom toxins, from the bloodstream into tissues, and that venom or exogenously applied NO, once in the bloodstream, can cause such inflammation.

### Inflammation facilitated exudation of albumin at the injection site

**Figure 3:**
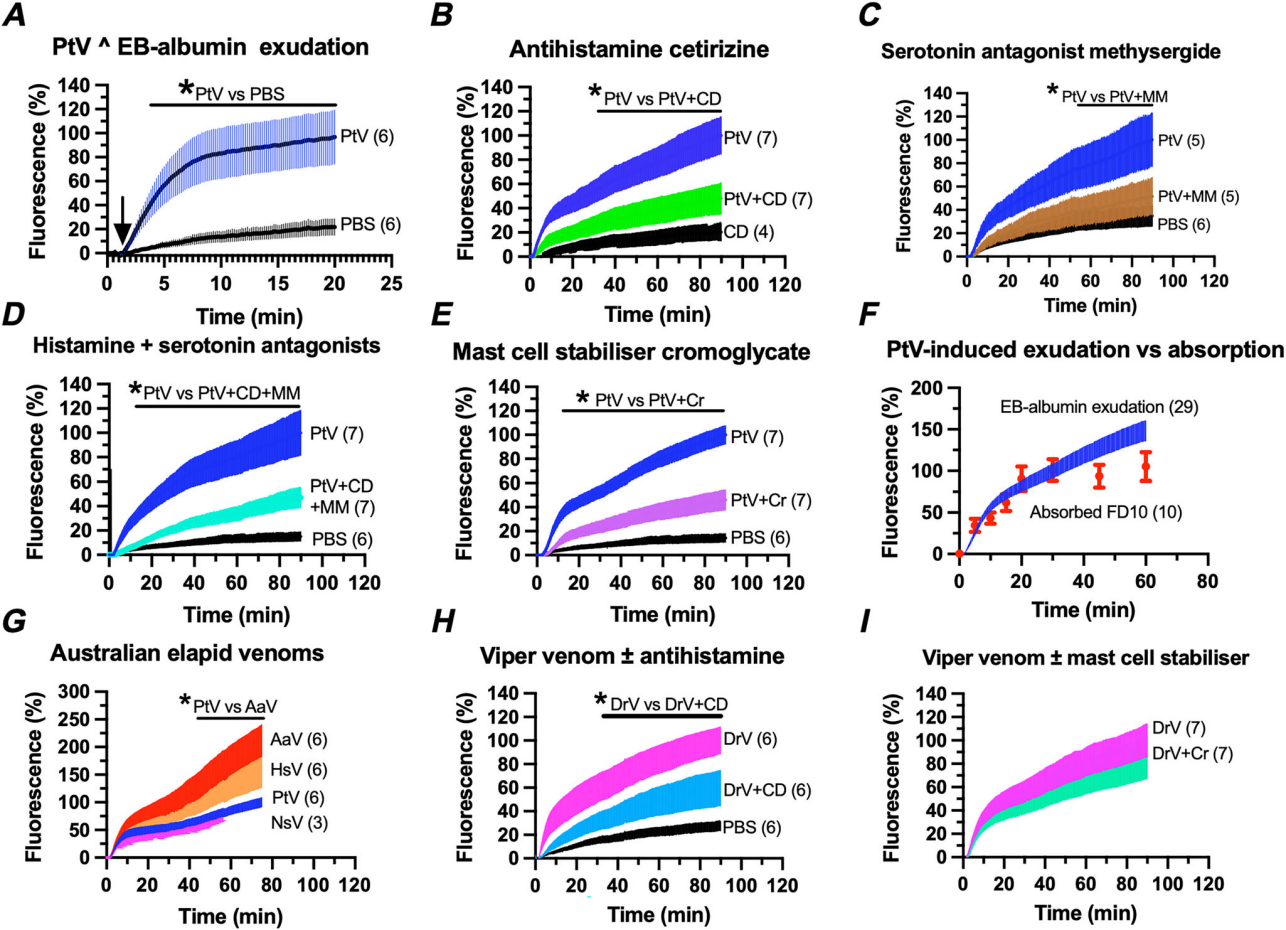
Venom‐induced increase in exudation of albumin at the hind skin site of injection *A*, exudation of Evans blue‐labelled albumin (EB‐albumin) from the bloodstream into the rat hind skin interstitium in response to subcutaneous injection of PtV in phosphate‐buffered saline (PBS) compared to injection of the same‐sized bolus (50 µl) of PBS. The arrow points to the onset of the responses after commencement of imaging, the latter starting 1–2 min after injection. *B–E*, effect of inhibitors antihistamine cetirizine dihydrochloride (CD), serotonin antagonist methysergide maleate (MM), CD + MM and mast cell stabiliser sodium cromoglycate (Cr) on exudation of EB‐albumin in response to injection of PtV compared to PtV without inhibitors. *F*, mean PtV‐induced EB‐albumin exudation due to injection of PtV compared to mean serum FD10, the latter in response to hind paw injection of FD10 + PtV (from Fig. *1A*). *G*, exudation of EB‐albumin in response to injection of elapid venoms AaV, HsV, PtV and NsV. *H*, injection of viper venom DrV compared to matched injection of DrV + CD. *I*, injection of viper venom DrV compared to matched injection of DrV + Cr. Concentrations used were PtV, AaV, NsV and HsV 0.05–0.15 mg/kg and DrV 0.1–0.2 mg/kg, CD 0.8 µg, Cr 2.5 µg and MM 8 µg. EB records were normalized to the averaged near peak value on each respective graph, except for *F* (30 min values) and *G* (peak PtV value). PBS was generally used as the control, except for *B* where PBS containing CD was used. Artifacts during the first 0–1 min were removed. Statistics – see legends Fig. 1. P‐values denoted by asterisks ranged as follows: *A*, 0.043–0.017 (Mann–Whitney test (MW)); *B*, 0.049–0.002 (ANOVA); *C*, 0.045–0.0081 (ANOVA); *D*, 0.047– < 0.001 (ANOVA); *E*, 0.047– < 0.001 (ANOVA); *F*, 0.727–0.125 (NS, MW); *G*, 0.049– < 0.001; *H*, 0.049–0.0045; *I*, 0.730–0.664 (NS, MW).

We measured local exudation of EB‐albumin from blood into the tissue interstitium at the injection site in the depilated posterior hind skin of anaesthetized rats using fluorescence (Methods, Procedures (c)). By imaging a wide hind skin region we simultaneously measured the response to injection of venom ± inhibitors at several non‐overlapping sites. As reported by van Helden et al. (2019) injection of PtV caused EB‐albumin exudation, which from the data of Fig. 3*A* increased exudation by 560% compared to the response to PBS (0–20 min AUC comparison from mean records). The measured response was activated with a delay of 1.5 min, but the total delay will be longer (∼3 min) as there was a delay of 1–2 min between injection and commencement of imaging.

Exudation of EB‐albumin was significantly reduced to 48%, 51%, 41% and 45%, respectively (*P = *0.0392, 0.0497, 0.0039 and < 0.001, 0–90 min AUC comparison; *t* test) of the PtV (0.1 mg/kg) response consequent to subcutaneous hind skin co‐injection of the antihistamine CD (Fig. 3*B*), the serotonin antagonist MM (Fig. 3*C*), CD + MM applied together (Fig. 3*D*) or the mast cell stabilizer Cr (Orr et al., 1971) (Fig. 3*E*). The profound effects of mast cell stabilization and antagonism of key mediators of mast cell‐released PIFs, such as histamine, suggest that PtV induced an acute vascular inflammatory response, with at least half resulting through the activation of mast cells.

PtV‐induced EB‐albumin exudation and PtV‐induced FD10 absorption (Fig. 1*A*) have kinetics that overlie each other up to ∼30 min after injection (Fig. 3*F*). Exudation then continues to rise whereas the absorption response plateaus. The similar kinetics of the initial response during absorption and exudation are consistent with both passing through IAPs, FD10 inward from the interstitium into the vasculature and EB‐albumin outward. The difference at longer times is most likely due to the slower clearance time of EB‐albumin from the interstitium compared to clearance of absorbed FD10 from blood.

### Exudation of albumin induced by several elapid venoms and a viper venom

Inflammation facilitated exudation of EB‐albumin caused by PtV was compared to the exudation response caused by three other quite different Australian elapid venoms, namely death adder (AaV), Stephen's banded snake (HsV) and tiger snake (NsV) for the same venom dose in each experiment. All caused substantial EB‐albumin exudation, with response waveforms exhibiting similar initial responses though followed by a further upswing particularly for AaV and HsV venoms (Fig. 3*G*). Comparison was made to Russell's viper (*D. russelii*) venom (DrV; Fig. 3*H* and *I*). DrV also markedly increased EB‐albumin exudation but showed an exudation profile that did not exhibit an upswing. Exudation following DrV was substantially inhibited (by 54%; AUC comparison) when the antihistamine CD was co‐injected (Fig. 3*H*). A notable difference to the action of PtV was the finding that the mast cell stabilizer Cr did not inhibit the viper venom‐induced inflammatory response (Fig. 3*I*).

### Inhibition of venom absorption by a pressure cuff and by pharmacological agents that target PIFs

**Figure 4:**
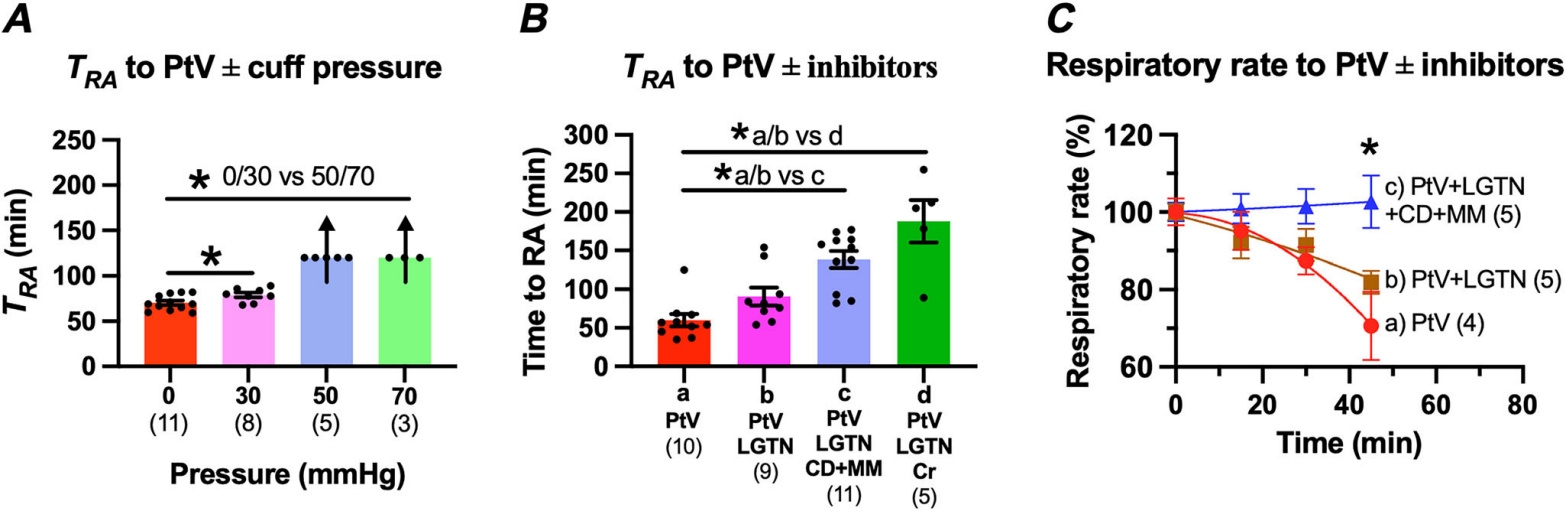
Time to respiratory arrest (*T*_RA_) to PtV with a pressure cuff or pharmacological inhibitors *A*, *T*_RA_ of anaesthetized rats consequent to hind paw injection of PtV at different cuff pressures (up arrows indicate animals survived for > 2 h at which time experiments stopped). *B*, *T*_RA_ consequent to injection of PtV; the same dose of PtV + leg application of nitric oxide (NO) donor LGTN (i.e. PtV + LGTN); this combination together with co‐injection of antihistamine cetirizine dihydrochloride (CD) + serotonin antagonist methysergide maleate (MM) (PtV + LGTN + CD + MM) or with mast cell stabiliser sodium cromoglycate (Cr) (PtV + LGTN + Cr). *C*, normalized respiratory rate for hind paw co‐injection PtV, PtV + LGTN and PtV + LGTN+ CD + MM (respective respiratory rates at *t* = 0 were 143 ± 5 (4), 142 ± 3 (5) and 133 ± 3 (5) cycles/min). Concentrations used were as follows: PtV: 1 mg/kg, CD: 0.8 µg, Cr: 2.5 µg, MM: 8 µg. Statistics: means ± SEM (animals); two‐way ANOVA with Tukey's multiple comparisons test; significance: *P*‐values denoted by asterisks were or ranged between *A*, 0.008– < 0.001; *B*, 0.0375–0.001 (*), a–b 0.355 (NS), c–d 0.363 (NS); *C*, at 45 min a–b 0.206 (NS), a–c < 0.001; b–c 0.005.

*P*_cuff_ studies, similar to those reported previously (van Helden et al., 2019) but using a wider range of pressures, were made on anaesthetized rats to investigate survival consequent to hind paw injection of PtV (1 mg/kg). A *P*_cuff_ of 30 mmHg had a small but significant effect on the time to respiratory arrest (*T*_RA_), increasing the mean control value of 69 ± 2.4 (11) to 79 ± 2.7 (8) min (*P = *0.0168; *t* test), whereas all rats survived for the 2 h duration of the experiments with a *P*_cuff_ of 50 or 70 mmHg (Fig. 4*A*).

Pharmacological studies were made on anaesthetized rats to investigate the effects of inhibiting the PtV‐induced inflammatory response. Lymphatic inhibition was maintained throughout these pharmacological experiments by topical leg application of a NO donor (Saul et al., 2011) with lanolin topically applied to the test limb for the controls. The *T*_RA_ for this experimental group was 60 ± 8 min (10) under control conditions (i.e. PtV at 1 mg/kg + lanolin) and 91 ± 12 min (9) with the addition of topical NO application to the test limb (i.e. PtV + LGTN). *T*_RA_ significantly increased to 139 ± 11 min (11) with the addition of PIF antagonists CD and MM to this protocol (i.e. PtV + LGTN + CD + MM) or to 188 ± 28 min (5) with the mast cell stabilizer Cr (i.e. PtV + LGTN + Cr) (Fig. 4*B*).

Respiratory rate, which is known to markedly slow in anaesthetized rats before respiratory arrest with PtV at 1 mg/kg (Saul et al., 2011), was also measured in some of these experiments. Slowing observed with PtV (1 mg/kg) also occurred with LGTN but was delayed following the application of inflammatory response inhibitors CD + MM over the time period shown (Fig. 4*C*). These data indicate that venom‐induced vascular inflammation opens a significant pathway for the absorption of venom toxins, and that partial inhibition of IAPs and resultant IFMA by agents such as an antihistamines prolongs survival, presumably by reducing venom load for the experimental conditions of this study.

### Antivenom

**Figure 5:**
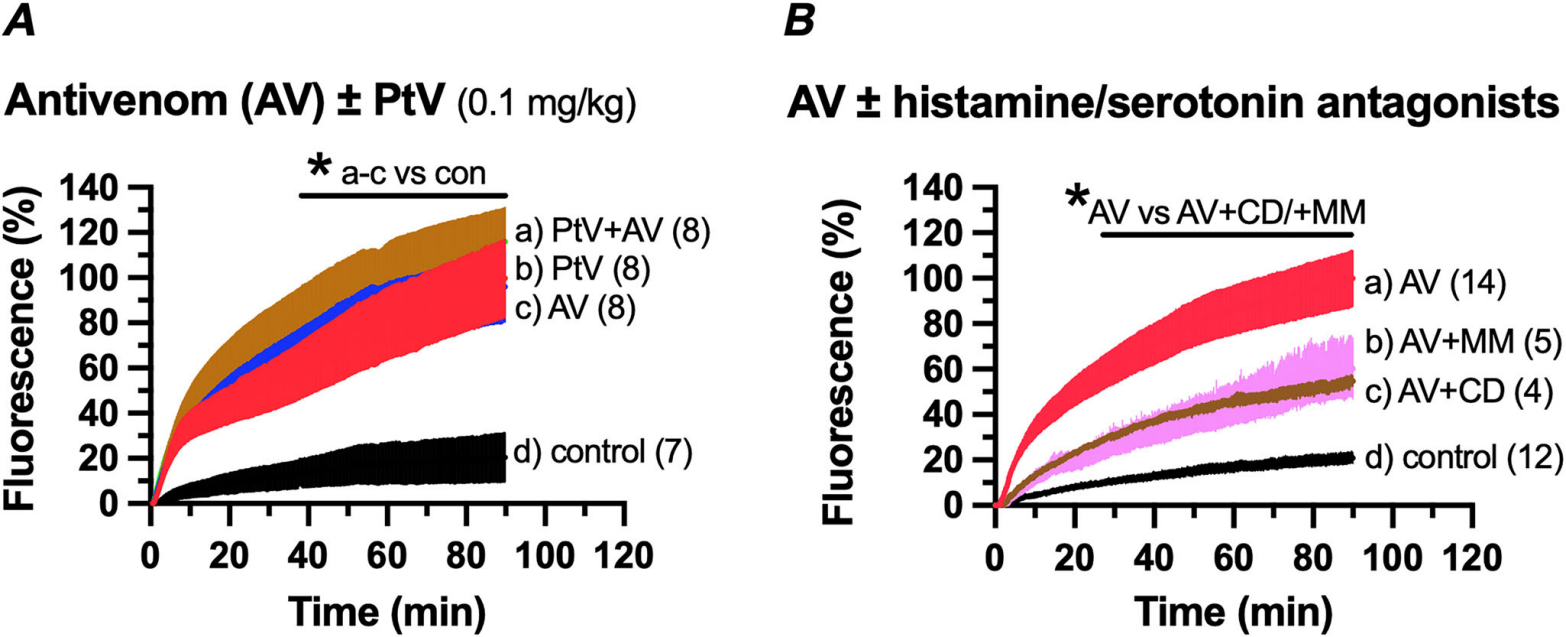
Antivenom actions *A*, vascular exudation of EB‐albumin from the bloodstream into the interstitium of posterior rat hind skin measured using fluorescence imaging. Recordings were made simultaneously consequent to subcutaneous rat hind skin injection of a phosphate‐buffered saline (PBS) bolus (∼50 µl) containing antivenom (AV; 4.5 units/kg; red), venom (PtV; 0.1 mg/kg; blue), venom + AV (brown) or control comprised PBS (*N* = 3) and saline (*N* = 4). *B*, comparison between hind skin injection of the same dose of AV as for *A*, AV + antihistamine cetirizine dihydrochloride (CD) (0.8 µg), AV + serotonin antagonist methysergide maleate (MM) (8 µg) or control (all PBS). Statistics: means ± SEM (number of animals); two‐way ANOVA with Tukey's multiple comparisons test; significance: *P*‐values denoted by asterisks ranged as follows: *A* a, b and c compared 0.995–0.605 (NS), a–c *versus* d 0.049– < 0.001; *B* a *versus* b and c 0.048–0.004, a *versus* d 0.043– < 0.001, b *versus* c 1.0000–0.9704 (NS).

Brown snake antivenom (AV; 4.5 units/kg) proved unsuccessful in inhibiting the PtV‐induced inflammatory response, as this commercially available AV itself caused an inflammatory response. This action was presumably due to contaminants such as IgE, the latter known to degranulate mast cells (Pearlman, 1999), as the response to the AV paralleled that caused by PtV (0.1 mg/kg) or PtV + AV (Fig. 5*A*). AV‐facilitated exudation was significantly inhibited by co‐injection of PIF antagonists, with both MM and CD resulting in an approximate halving of EB‐albumin exudation (Fig. 5*B*).

### Macromolecular absorption compared to the underlying permeability increase

**Figure 6:**
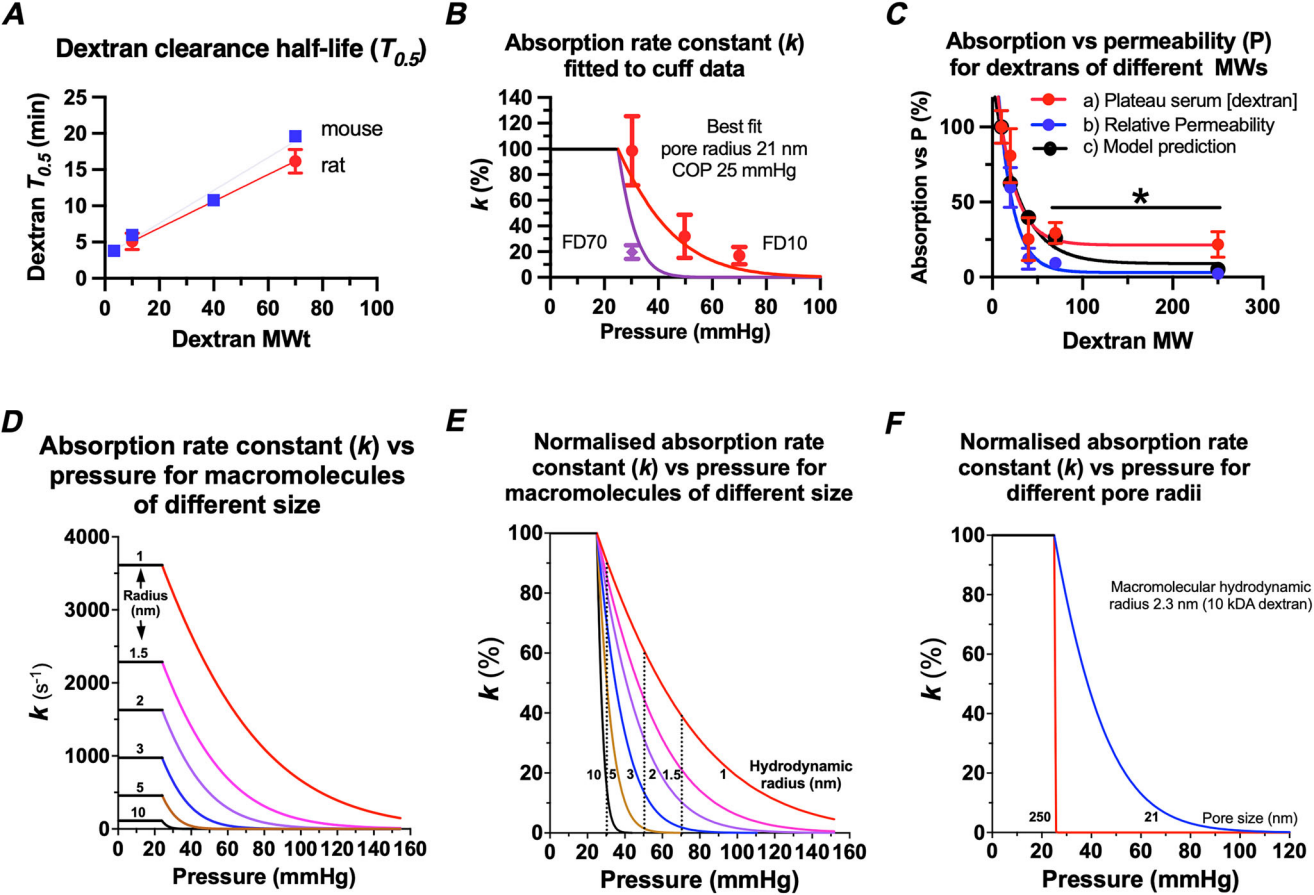
Macromolecular absorption and clearance during vascular inflammation *A*, dextran clearance half‐times (*T*_0.5_) for molecular weights (MWs) up to a 70 kDa dextran from rat vasculature compared to data derived from published mouse studies (Dreher et al., 2006). *B*, determination of the size of the inflammatory‐activated pore (IAP) using best‐fit analysis (Methods – Modelling) of simulated absorption rate constant *k* (see *D* and *E*) *versus* venular pressure for 10 and 70 kDa dextrans (respective hydrodynamic radii 2.3 and 5.5 nm) when constrained to the experimental pressure cuff data (Appendix: Absorption at positive hydraulic pressures). Flat region is the range where venular pressure is less than colloid osmotic pressure. *C*, serum FD absorption levels as a function of MW (from Fig. 2*F*; red) compared to the calculated relative dextran venular permeability, which is equivalent to the relative absorption rate constant *k*_in_ (blue; Results: Macromolecular absorption compared to the underlying permeability increase) and the model‐based absorption rate constant (black), calculated using the plateau levels from *D*. The curves are exponential fits (Prism) of each dataset. *D* and *E*, simulated absorption rate constant *k* (*D*) or in normalized form (*E*) as based on eqns (8) and (10) (Appendix convection–diffusion equations) for a 21 nm IAP radius plotted against venular pressure for a range of macromolecular hydrodynamic radii (NB: dotted reference lines in *E* really are vertical). *F*, comparison of normalized rate constant plotted as a function of venular pressure for a 10 kDa dextran when passing through a pore of radius 250 nm compared to the 21 nm pore. Statistics: (*A*–*C*) means ± SEM; *A*, *N* = 3/point for both FD10 and FD70 data, mean for mouse data (*N* = 3/point); *B*, *N* = 3–10 for FD10 and 6–8 for FD70; *B*, data used to fit pore size (see text); *C*, *N* = 5–8, multiple unpaired *t* tests, significance: *P*‐value for a–b denoted by asterisk ranged between 0.040 and 0.022.

Macromolecular absorption achieves a plateau, which reflects an apparent steady‐state situation where absorption matches clearance from the circulation. Using our measurements of plateau dextran absorption (Fig. 2*F* serum data) and clearance (Fig. *6A*) we calculated the relative permeabilities for the 10–250 kDa dextran range by simple first‐order kinetics (Appendix – Calculation of absorption permeability from dextran uptake and clearance: eqns (12)–(16)). Comparison of these data‐based calculations indicates that the decline in dextran permeability underestimates the decline in the vascular‐absorbed FD measured from the serum plateau levels  (Fig. 6*C*).

### Modelling absorption

Theoretical modelling indicates that macromolecules can diffuse across the microvasculature in both directions (Curry & Michel, 2025; Nagy et al., 2012; Rippe & Haraldsson, 1994). We modelled the absorption of macromolecules in venom‐inflamed tissues based on such theoretical background (Rippe & Haraldsson, 1994) (Appendix – Convection–diffusion equations: eqns (1)–(10)). This allowed simulations of our experimental findings providing insight into the ‘battle’ between vascular inflow of macromolecules by diffusion against venular pressure‐related outflow of plasma. The results are shown in a series of plots comparing macromolecular absorption rate constant *k* to venular pressure for different‐sized macromolecules over a molecular weight and consequent size range that approximately match known venom toxins. Snake venom toxins have molecular weights typically ranging from 6 to 250 kDa (Markland, 1998), with corresponding calculated hydrodynamic radii (*r*_0_) typically of 1.3–6 nm, a range encompassed by our modelling (Fig. 6*B–E*).

Under vascular inflamed conditions macromolecular permeation dominantly occurs through IAPs and not carrier‐mediated transport across vascular endothelial cells, consistent with the marked dependence of absorption on venous/venular pressure. Blood vessels near or above heart level exhibit venular pressures typically less than the osmotic pressure (Brenner et al., 1972; House & Johnson, 1986). This allows ready diffusion‐based absorption or exudation of macromolecules at higher levels in the interstitium or vasculature, respectively, as under these conditions there is no outward pressure‐induced convection of plasma. However for vessels below heart level, at venular pressures exceeding blood osmotic pressure plasma with its solutes now exudate through convection from venules through the IAPs into the tissues. This convection‐based outward flow of plasma increasingly opposes inward diffusion (i.e. absorption) of macromolecules, an action that has a greater effect on larger compared to smaller macromolecules (Fig. 6*B–E*).

A key component of these simulations is the size of the IAPs. We determined the average IAP size under inflamed conditions using our pressure cuff data by least‐squares fitting (Methods – Modelling) of the model‐based simulations to the cuff pressure (*P*_cuff_) data determined at 30, 50 and 70 mmHg for FD10 (hydrodynamic radius 2.3 nm) and 30 mmHg for FD70 (*r*_0_ 5.8 nm). The best log‐linear least‐squares fit corresponded to a mean IAP radius of 21 nm (95% CI 18–24 nm) (Fig. 6*B*). This value is similar to the 24 nm radius pore size reported for the small (< 5%) contingent of large pores present under non‐inflamed conditions (Levick & Michel, 2010). Such pore sizes are much larger than those of any known venom toxin, the largest reported being ∼300 kDa (Markland, 1998), which will have a hydrodynamic radius of < 10 nm, well below that of these inflamed pores. Therefore large macromolecules, including all snake venom toxins, should permeate through these IAPs.

## Discussion

Venomous snakes have evolved to be highly successful predators through an arsenal of adaptations. One of these is their venomous bite, causing local acute vascular inflammation through opening of large IAPs (see Introduction). By experiment we show that IAPs underlie IFMA, a mechanism that underlies direct venom absorption of interstitial macromolecules into the bloodstream (Abstract Fig. *A* and *B*).

Snake envenomation induces inflammation through components in their venoms and through factors associated with the injection itself. This latter mechanism seemed to be location dependent, being observed in response to subcutaneous injection into the rat hind paw but not to similar injection into the hind skin. This response was relatively smaller than the venom‐induced inflammation and was transient, lasting about an hour, and was not obviously due to the fluorescent dextran. This difference has not been further investigated, but factors such as the hind paw being a more restricted space compared to that of the hind skin need to be considered.

Our studies primarily focused on one venom (elapid venom, *P. textilis –* PtV). The resulting data together with modelling allowed reasonable interpretation of the actions of other selected venoms made by imaging albumin exudation. Integral to this is the finding that PtV‐induced inflammation opens IAPs that simultaneously allow plasma exudation and absorption of interstitial macromolecules, most dominantly for vasculature near heart level where the hydrostatic pressure is low. Therefore under these conditions inflammation‐induced plasma albumin exudation inversely mirrors the absorption of macromolecules dominantly present in the interstitium. This is likely to be the case regardless of the inflammatory activator providing acute vascular inflammation is induced.

### Permeability of different‐sized macromolecules and the role of the lymphatics

Macromolecules that have the potential to be absorbed include the dextrans of our study and all known snake venom toxins, given that the 21 nm pore radius of IAPs is large compared to these molecules. We investigated the relative permeability of dextrans with molecular weights of 10, 20, 40, 70 and 250, using vascular absorption and ear exudation measurements, with the results compared to modelling, which exhibited similar profiles (Fig. 2*F* and Fig. 6*C*). The largest macromolecule (i.e. FD250), which has a large *r*_0_ of about 11 nm, was absorbed at a substantially diminished level compared to FD10 (*r*_0_ 2.3 nm). In contrast the *r*_0_ of very large venom toxins such as Pseutarin C, which has a molecular weight of ∼250 kDa (Rao & Kini, 2002), has a markedly smaller *r*_0_ (∼5 nm) than that of the 250 kDa dextran and so will be more readily absorbed through IAPs. The smaller *r*_0_ is a consequence of the compact structure of venom toxins compared to linearly arranged dextrans, the latter coiling in free solution (Armstrong et al., 2004).

In cases where the vascular system is near heart level, such as in our rodent studies, direct vascular absorption is the dominant pathway for venom (PtV) absorption compared to that provided by the lymphatics at least over the first 2 h of our study, although sheep studies indicate this dominance is lost over longer times (Paniagua et al., 2012). The minimal role of lymphatics measured 2 h after envenomation makes it unlikely that lymphatic uptake at this time influences net absorption of dextrans. However it would explain why peak levels of the largest dextrans in the serum were higher than that predicted from the model (Fig. 6*C*). This finding might also be explained if the actual exudation rate (*k*_out_) is slower than that predicted from the half‐time of vascular clearance of macromolecules (Fig. 6*A*), which also includes other clearance mechanisms.

A confounding issue raised by our experiments relates to the topical application of leg‐applied LGTN ointment and resultant NO, which has been shown to be potentially beneficial as a first aid through inhibiting lymphatic absorption (Saul et al., 2011; van Helden et al., 2014). However the current experiments demonstrate that LGTN application caused a marked increase in acute vascular inflammation, as measured by increased dextran absorption consequent to hind paw injection of FD10 in the absence of venom (Fig. 2*H*). We investigated this conundrum by repeating the same experiment in the presence of venom, finding that under these conditions exogenous NO did not further increase vascular inflammation (Fig. 2*I*). The finding that venom and NO both induce acute vascular inflammation opens the possibility that, just as NO inhibits lymphatic function, venom itself may do the same. This might explain observations such as lymphatics having no role in venom absorption over at least the first hour after envenomation in sheep (Paniagua et al., 2012). It is also consistent with the observation that the initial enhancement of venom absorption by lymphatics in anaesthetized rats observed in a previous study reduced to a similar plateau level to that when lymphatics were inhibited (see figure 2 in van Helden et al., 2019).

### The response to other venoms and the potential of haemorrhage being a confounding factor

We compared several venoms, including three other quite different elapid venoms and a viper venom to PtV action, by measuring albumin exudation in response to subcutaneous venom injection. This method was used as it represented a direct measurement of IAPs, and comparative studies could be made in the same animal. The cytotoxic viper venom DrV introduced the possibility of substantial haemorrhage, which could confound interpretation. Transvascular ‘pores’ large enough to pass red blood cells will have a radius of at least 250 nm (Deplaine et al., 2011), and such ‘pores’ would facilitate substantial exudation of plasma. Notably at or above heart level such pores will allow venom entry approximately proportional to the ∼140 times larger cross‐sectional area compared to that of an IAP. However the outward flow of plasma through these pathways is so large to completely inhibit venom absorption through these pores by a pressure cuff or for bites even a small distance below heart level. Based on the hydrostatic pressure exerted by a 1 cm column of blood equating to ∼0.7 mmHg, maintaining a bite site about 20 cm below heart level will move the venular pressure to about 25 mmHg, assuming a venular pressure of 10–15 mmHg at heart level (House & Johnson, 1986), and once this pressure is reached then even the slightest increase in venular pressure will prevent absorption through these extremely large ‘pores’ (Fig. 6*F*). IAPs should function normally despite the simultaneous presence of haemorrhage‐associated ‘pores’, with direct vascular venom absorption simultaneously reduced by maintaining the site below heart level, but to a lesser extent.

Evidence against haemorrhage‐associated pores subserving a substantial role in DrV‐induced albumin exudation during the relatively short 1–2 h time course of our experiments is provided by several findings. Haemorrhage onsets over much longer time period than the acute vascular inflammatory response caused by opening of IAPs. The estimated haemorrhage onset time period based on coagulopathy measured in humans for DrV is 0.5–12 h (Kularatne, 2003; Priyankara et al., 2022), yet the DrV albumin exudation response commenced almost immediately (Fig. 3*H*). Importantly we show that both PtV and DrV induce albumin exudation responses with profiles that are very similar and where a substantial component of the response is inflammation mediated, given that both responses were inhibited by ∼50% by an antihistamine (Fig. 3*B* and *H*). This finding is consistent with opening of IAPs during our 1–2 h experiments and not haemorrhage‐induced pores where inflammation does not have a primary role.

Together these findings indicate that under the conditions of our experiment Russell's viper venom DrV acts in an analogous manner to the inflammatory response caused by elapid venom PtV. The exudation responses to three other quite different elapid venoms (i.e. from death adder, Stephen's banded and tiger snake) all exhibited initial responses similar to that caused by PtV. However there was enhanced exudation at longer times (i.e. > 30 min), particularly for death adder (AaV) and Stephen's banded snake (HsV). This enhancement is yet to be investigated.

### Differences in initiation of elapid and viper venom‐induced inflammation

As for contaminants in the PtV antivenom (Fig. 5) and many other known activators (Galli et al., 2013; Theoharides et al., 2023) elapid venom (PtV) caused acute vascular inflammation by activating mast cells. In contrast Russell's viper venom utilized mechanisms that did not obviously involve mast cells. Elapids are short‐fanged and so inject venom subcutaneously, whereas vipers are long‐fanged, injecting venom into deeper tissue regions. This difference in actions between the elapid and viper venoms needs to be further investigated, as it may represent an evolutionary trait given that mast cells are prolific in skin regions compared to deeper regions (Fong & Crane, 2023).

### Role of venom‐induced inflammation in movement of toxins from the vasculature into tissues

Snake envenomation not only depends on toxin absorption into the bloodstream at the bite site but also on toxin exudation into the tissues once in the bloodstream (Abstract Fig. *C*). This latter process also requires inflammatory factors. These are likely to be present in absorbed venom toxins that being in the circulation increase inflammation widely throughout the body. Such action markedly increases exudation of venom toxins into tissues and is essential for permeation of larger toxins through vessels with continuous epithelia, as without inflammation macromolecules of *r*_0_ ≳ 5 nm (e.g. FD70) do not substantially exudate from the bloodstream of such vessels (Egawa et al., 2013; Mayhan, 1994). Inflammation in the circulation will also facilitate other macromolecules, including AV antibodies (e.g. *r*_0_ for IgG is ∼5.5 nm), in blood to exudate into tissues through IAPs. There are various mechanisms by which intravascular venoms could induce inflammation, including venom‐associated PIFs and/or activation of blood‐borne PIF‐containing cells such as basophils (MacGlashan, 1993), macrophages, monocytes and lymphocytes (Zwadlo‐Klarwasser et al., 1994).

### Therapeutic implications

Although the mechanical snakebite first‐aid procedure termed ‘pressure bandaging with immobilization’ (PBI) is known to inhibit lymphatic transport (Howarth et al., 1994), it will also inhibit direct vascular absorption of venom toxins. Our studies indicate that PBI will be especially effective in inhibiting such absorption for larger venom toxins but less so for smaller toxins. From modelling (Fig. 6*E*) PBI applied at 50–70 mmHg should inhibit > 80% of direct vascular absorption of lethal brown snake venom toxins such as Textilotoxin (Tyler et al., 1987) or Pseutarin C (Rao & Kini, 2002), toxins that are relatively large with hydrodynamic radii > 3.5 nm. However it will be limited in its effectiveness in inhibiting direct vascular absorption of small venom toxins, and therefore its value will be impaired for bites from snakes where three‐finger toxins and dendrotoxin (*r*_0_ 1.0–1.5 nm) are dominant unless the toxins have larger *r*_0_ by functioning as multimers. For example a toxin with a *r*_0_ of about 1.3 will be about 50% inhibited by a pressure bandage at 50 mmHg, and this will worsen for even smaller toxins (Fig. 6*E*).

An alternative mechanical approach that may circumvent limited effectiveness of PBI for such venom toxins is the pressure pad technique (Anker et al., 1982; Tun et al., 1995; Tun et al., 2000), which used together with immobilization has been shown to inhibit absorption of even smaller molecules. It involves using a tight sprain‐like bandage, preferably over the same pressure range as PBI, to compress a pad over the bite site, with the pad exerting higher local pressure due to its smaller application area. Thus in the above example an increase in pad pressure over the bite site will proportionally inhibit the level of direct venom absorption into the vasculature, with pad pressures of 70, 90 or 110 mmHg causing approximately 60%, 75% or 85% inhibition, respectively, for a toxin with a hydrodynamic radius of 1 nm (Fig. 6*E*).

In cases of haemorrhage at the bite site the potentially large absorption provides some impetus to act quickly to prevent such influx. Given the extreme venular pressure dependence for such absorption (Fig. 6*F*) a recourse might be to immediately maintain the site 20 cm or more below heart level, if possible, until the application of a pressure bandage (also see Discussion – The response to other venoms and the potential of haemorrhage being a confounding factor). Hydrostatic pressure will also inhibit vascular absorption through IAPs, but higher pressures (i.e. larger distances below heart level) would be required to provide substantial inhibition .

Pharmacological approaches to snakebite first aid are also of interest in reducing venom load (Saul et al., 2011) or cytotoxic damage (Bittenbinder et al., 2024). Our research introduces new pharmacological directions for reducing direct vascular absorption of venom toxins, which build on inhibiting the lymphatic pathway (Saul et al., 2011; van Helden et al., 2014; van Helden et al., 2019). For example an H1 histamine antagonist reduced direct vascular absorption by about 50% for both the primary elapid (PtV) and viper venom (DrV) of our studies. Therefore the administration of H1 antihistamines may be of value in reducing venom load in snakebite victims though immobility would need to be maintained. Further investigations need to be made to determine the mechanisms underlying the residual 40%–50% of the inflammatory response.

### Inflammation‐facilitated macromolecular absorption

IFMA is unlikely to simply be a latent trait there for the convenience of snakes and other venomous creatures whose venoms cause inflammation. Rather it is likely to be an important albeit understudied physiological mechanism that operates in parallel with the lymphatic system for clearing unwanted interstitial macromolecules. IAPs will pass macromolecules of considerable size depending on their relative hindrance (Deen, 1987), the pores having a mean radius of about 21 nm. Therefore IAPs and resultant IFMA will subserve a clearing function dependent on the size of the macromolecule and concentration gradient between the interstitium and blood. There will also be some clearance of macromolecules under non‐inflamed conditions but, as indicated by our experiments, this is much smaller large pores representing less than 5% of the resident pore population (Levick & Michel, 2010).

IFMA is likely to have a role in clearance of cellular breakdown products such as DNA fragments, oligonucleotides, peptides, fatty acids, nucleotides and amino acids, which have hydrodynamic radii typically in the range 0.5–10 nm. In this regard IFMA may function in body processes such as during the inflammatory phase of wound healing where there is acute long‐lasting vascular inflammation (Teller & White, 2009). This mechanism would improve clearance of cellular breakdown products in wound regions near heart level where venous/venular pressures are low. However wound‐associated IFMA would be compromised in the lower limbs of humans when standing due to high hydrostatic pressure reducing or preventing absorption, a circumstance that would not inhibit lymphatic uptake, which is protected against hydrostatic pressure by regularly occurring valves (Olszewski & Engeset, 1980). Thus wound‐associated IFMA may be a contributing factor underlying the empirical finding that maintaining leg wounds near heart level by raising the limb improves healing.

## Conclusions

In overview our studies highlight the importance of IFMA as a mechanism that functions in parallel with the lymphatic system to absorb and clear cellular debris or exogenous interstitial macromolecules. Venomous snakes have evolved to utilize this mechanism, with their venoms causing acute vascular inflammation and opening of IAPs, thereby facilitating venom toxin absorption through IFMA and promoting toxin exudation into tissues by also inducing inflammation once in the bloodstream. These strategies markedly enhance envenomation and, therefore, the capture of prey such as rodents, reptiles, amphibia and birds, organisms that all have permeability‐increasing factor–associated mechanisms (Baccari et al., 2011), and participate in making venomous snakes extremely dangerous to humans. The findings present new possibilities for improving first aid for snakebite. These include procedural optimization of existing mechanical pressure‐based first‐aid methodologies based on venom toxin characteristics and herald new pharmacological strategies that target the acute vascular inflammatory response.

## Additional information

## Competing interests

The authors declare no competing interests.

## Author contributions

Design of the study: D.F.v.H. Experimentation: P.J.D. Modelling: D.R.L. Statistics: S.A.M. Imaging: J.H. Project collaboration: N.S., D.J.B., K.C., T.A.H. Analysis and writing of the manuscript: D.v.H. and D.R.L. Critical review of the manuscript: N.S., S.M., D.B., K.C., P. J. and T.A.H.

## Funding

This work was supported by the Translational Australian Clinical Toxicology: John Holdsworth (Grant No. G1701330 awarded to D. F. van Helden).

## Supporting information


Peer Review History

## Data Availability

The conclusions of this study are based on data presented in the text. Additional information is available from the corresponding author on reasonable request.

## Convection–diffusion equations

Theoretical modelling has long indicated that macromolecules can diffuse across the microvasculature (Rippe & Haraldsson, 1994). We model the absorption of macromolecules in venom‐inflamed tissues based on this theoretical background. We consider a situation where macromolecules diffuse from the interstitium into the bloodstream through IAPs, which are large solvent‐filled transvascular pores in the endothelium of venules (Egawa et al., 2013; Levick & Michel, 2010; Majno & Palade, 1961; Rippe & Haraldsson, 1994). At positive venular pressures (i.e. *ΔP*>0, where *ΔP* includes hydraulic and osmotic components, see eqn (3)), there is outward convective (i.e. bulk) flow of solvent that under inflamed conditions becomes an outward convective flow of plasma, the latter including macromolecules (i.e. solutes) such as albumin. Consistent with the ‘Starling Principle’ outward convective flow will be proportional to *ΔP* when the hydraulic component (*P*_v_) exceeds ∼25 mmHg, approximately equivalent to the osmotic pressure in humans and rats (Brenner et al., 1972; Morissette, 1977).

At *ΔP* below this osmotic pressure and under steady‐state conditions convection through IAPs deviates from the ‘Starling Principle’ and asymptotes to zero rather than going negative (Levick & Michel, 2010). This will be the case for venules near heart level, given that at heart level venular pressure is on average ∼15 mmHg for humans (House & Johnson, 1986) and likely even less for rats. In this case *ΔP* will be effectively 0 mmHg, a circumstance when there is both inward diffusion of interstitial macromolecules such as venom toxins and outward diffusion of blood solute macromolecules such as albumin (Fig. 6*B*). There will be little competition between such inward and outward diffusion given the large size of IAPs. At positive *ΔP* inward diffusion is now opposed by outward convective flow of plasma, with the outward flow increasing as *P*_v_ increases (Fig. 6*B*). We calculate the results of this competition using equations derived from a more general analysis reviewed by Rippe and Haraldson (1994). The resultant model presented here predicts that at low *ΔP* there is still substantial inward diffusion of molecules (i.e. absorption), which is proportionally greater for smaller molecules, with absorption decreasing as *P*_v_ increases for all molecules.

To understand the absorption of macromolecules (dextrans or venom toxins) in venules we consider the diffusion of solvent molecules (radius *r*_o_) within a single pore (radius *r*_p_) into the venule in which there is a convective flow of solvent out of the venule. We start with the non‐linear global convection–diffusion equation (the Patlak equation), describing the flux of solute, *J*_s_, across a membrane where there exists a solvent flow *J*_v_ taken from Rippe and Haraldsson (1994):

(1)
$$J_{s}=J_{v}\;\left(1-\sigma \right)\frac{C_{p}-C_{i}e^{-Pe}}{1-e^{-Pe}}$$

where *C*_p_ and *C*_i_ are the solute concentrations in the lumen and interstitium, respectively, and:

(2)
$$J_{v}=L_{p}\;S\Delta P$$

where *S* is the surface area, *L*_p_ is the hydraulic conductance and *ΔP* is given by the following:

(3)
$$\Delta P=\Delta P_{v}-\sigma \Delta \Pi$$

where *ΔP*_v_ and *Δ∏* are the differences in hydraulic and osmotic pressures between the plasma and interstitium. σ is the reflection coefficient of the membrane, which was calculated using the Renkin equation (Methods – *Modelling*). We assume that the dependence of *J*_v_ on Δ*P*_v_ is the same as the steady‐state dependence of *J*_v_ on capillary pressure (*ΔP*_c_), measured by Levick and Michel (2010), that showed a linear relationship between *ΔP*_c_ and *J*_v_ for *ΔP*_v_ above ∼25 mmHg, which asymptotes to zero *J*_v_ at lower *ΔP*_c_. Thus we infer that for *ΔP*_v_ above 25 mmHg, *ΔP ≅ ΔP*_v_ – 25 mmHg and is approximately zero at lower *ΔP*_v_.

The relative contributions of diffusion and convection to solute flow can be assessed from the modified Peclet number, *Pe*, which is large in cases where solute flow is mainly due to convection and small when mainly due to diffusion.

(4)
$$Pe=J_{v}\left(1-\sigma \right)\Delta x/D_{s}S^{\prime }\;$$

*S’* is the restricted area available for solute in the pore (*π(r*_p_ *−r*_o_)^2^), *D_s_* is the solute diffusion constant and *Δx* is the membrane thickness. From eqns (1) and (2):

(5)
$$J_{s}=L_{p}\;S\Delta P\frac{C_{p}-C_{i}e^{-Pe}}{1-e^{-Pe}}$$

Given that:

(6)
$$D_{s}=\frac{kT}{6\pi \eta r_{o}}\;$$

and

(7)
$$L_{p}S=\frac{\pi r_{p}^{4}}{8\eta \Delta x}\;$$

Substituting *D*_s_ from eqn (6), *L*_p_ *S* from eqn (7) and *J*_v_ from eqn (2) into eqn (4) we can derive the Peclet number for solute molecules (radius *r*_0_) in a pore (radius *r*_p_):

(8)
$$Pe=0.75\left(1-\;\sigma \;\right)\Delta P\pi r_{o}r_{p}^{4}/kT{\left(r_{p}-r_{o}\right)}^{2}$$

Because the molecules are initially added to the interstitial tissue *C*_P = _0. The initial rate of solute absorption from eqn (5) is then:

(9)
$$J_{s}=L_{p}\;S\Delta P\frac{-C_{i}e^{-Pe}}{1-e^{-Pe}}$$

The relative ΔP dependence of solute flow for a given value of $L_{p}S$ is then:

(10)
$$J_{s}=\mathrm{Const}*\left(1-\;\sigma \;\right)\Delta P\frac{-e^{-Pe}}{1-e^{-Pe}}$$

Equation 10, which depends on the Peclet number (eqn (8)), was used to simulate the relative absorption rate as a function of venular pressure for a range of dextran sizes, with the results shown both as simulated and in normalized form (Fig. 6*D* and *E*).

### Calculation of the reflection coefficient (σ)

The solute reflection coefficient was determined for permeation through a pore using the Renkin equation (for hindered transport) (Deen, 1987; Renkin, 1954) which is:

(11)
$$\sigma ={\left(1-{\left(1-\lambda \right)}^{2}\right)}^{2}\;$$

where *λ = r*_o_ */r*_p,_ with *r*_o_ the hydrodynamic radius of the permeating solute and *r*_p_ the pore radius.

A detailed analysis of different approaches to determine σ concluded that the Renkin equation is reasonably accurate when used for circumstances where *λ < *0.4 (Deen, 1987). This applies to nearly all simulations in our study but may underestimate σ in the case where λ was ∼0.5.

## Absorption at positive hydraulic pressures

Some experiments were conducted with a pressure cuff, which had a spin off, in that it provided a means for determining the mean size of IAPs. To model the effect of changes in *P*_v_ by a pressure cuff on diffusive absorption of macromolecules we assume that *P*_v_ is near that of the *P*_cuff_ when *P*_cuff_ exceeds physiological *P*_v_. Equating *P*_v_ with *P*_cuff_ is made because the considerable venular leak under inflamed conditions brings venular pressure closer to that of the veins. We fitted simulated absorption rate constant–venular pressure curves to optimally fit *P*_cuff_ for FD10 and FD70, whose hydrodynamic radii are 2.3 and 5.8 nm (Sigma‐Aldrich). Simulated absorption rate constant values and experimental measures of absorption for various cuff pressures were normalized to their respective values at 0 mmHg pressure (i.e. *P*_cuff_ *_= _* 0; Fig. 6*B*) to overcome the need to correct absorption for blood clearance rates (Fig. 6*A*). The normalized experimental cuff data averaged using the 45 and 60 min time points were 98 ± 27 (5), 32 ± 17 (4) and 17 ± 7% (3) for hind paw co‐injection of FD10 + PtV (1/mg/kg) at *P*_cuff_ 30, 50 and 70 mmHg, respectively (Fig. 1*G*), and 20 ± 5% (6) for FD70 + PtV (1/mg/kg) at 30 mmHg (Fig. 1*H*). The unknown variables in the simulation were the pore radius and the colloid osmotic pressure (COP), which, using least‐squares analysis fitted with MATLAB routines, were determined to be 21 nm (95% CIs 18–24 nm) and 25 mmHg (95% CIs 17–32 nm), respectively (Methods–Statistical analysis and modelling; Fig. 6*B*). These values were used to generate the absorption rate *versus P*_v_ curves for macromolecules of different hydrodynamic radii (Fig. 6*D* and *E*) and for comparison with absorption through an assumed haemorrhage‐associated ‘pore’ (radius 250 nm; Fig. 6*F*).

## Calculation of absorption permeability from dextran uptake and clearance

The steady‐state dextran concentration (FD_SS_) in the serum is related to the concentration in the footpad (FD_FP_) by:

(12)
$$FD_{\mathrm{SS}}=FD_{\mathrm{FP}}\;\times k_{\mathrm{in}}/k_{\mathrm{out}}$$

*k*_in_ is the absorption rate constant through venular pores, which is proportional to their dextran permeability.

(13)
$$k_{\mathrm{in}}\propto \mathrm{permeability}$$

*k*_out_ is the clearance rate constant, which is inversely proportional to clearance half‐time (*T*_0.5_; Fig. 6*A*).

(14)
$$k_{\mathrm{out}}\propto 1/T_{0.5}$$

FD_SS_ is proportional to the measured plateau FITC‐dextran level (Fig. 6*C*), and provided FD_FP_ is the same in each experiment from eqn (12) we can determine that:

(15)
$$\mathrm{dextran}\;\mathrm{permeability}\;\propto FD_{\mathrm{SS}}/T_{0.5}\;\left(i.e.\;k\mathrm{in}\right)$$

where *T*_0.5_ is calculated from the linear fit to the clearance data presented in Fig. 6*A*, resulting in the relationship:

(16)
$$T_{0.5}=0.18\times \mathrm{MW}+3.3$$
