# Supplementary material for: Inflammation induced by snake venoms optimizes envenomation
Source: J Physiol. 2026 Jun 8;604(14):5831–52. doi: 10.1113/JP290494 (PMC13370727; doi:10.1113/JP290494)
Supplement: Supplementary file 1 — Peer Review History [file TJP-604-5831-s001.pdf]

The Journal of Physiology

Inflammation induced by snake venoms optimises envenomation

Dirk Ferdinand van Helden, Neil James Spratt, Peter John Dosen, Sally Anne McFadden, John Holdsworth, Daniel J Beard, Kirsten Coupland, Phillip Jobling, Todd A Hardy, and Derek Laver
DOI: 10.1113/JP290494

Corresponding author(s): Dirk van Helden (dirk.vanhelden@newcastle.edu.au)

The referees have opted to remain anonymous.

Review Timeline:

Submission Date:	10-Nov-2025
Editorial Decision:	13-Feb-2026
Revision Received:	10-Mar-2026
Editorial Decision:	23-Mar-2026
Revision Received:	24-Apr-2026
Accepted:	18-May-2026

Senior Editor: Vaughan Macefield

Reviewing Editor: Sophie Møller

Transaction Report:

(Note: With the exception of the correction of typographical or spelling errors that could be a source of ambiguity, letters and reports are not edited. Depending on transfer agreements, referee reports obtained elsewhere may or may not be included in this compilation. Referee reports are anonymous unless the Referee chooses to sign their reports.)

Dear Dr van Helden,

Re: JP-RP-2025-290494 "**Bites from venomous snakes target an immune system response for optimal envenomation**" by Dirk Ferdinand van Helden, Neil Spratt, Peter John Dosen, Sally Anne McFadden, John Holdsworth, Daniel J Beard, Kirsten Coupland, Phillip Jobling, Todd A Hardy, and Derek Laver

Thank you for submitting your manuscript to The Journal of Physiology. It has been assessed by a Reviewing Editor and by 1 Referee and relevant feedback from their reports is copied below for your information. We regret to inform you that your manuscript is not considered acceptable for publication in The Journal of Physiology.

However, as the reviewers highlighted a number of strengths in your manuscript, we would like to suggest that you consider submitting your manuscript to our sister journal (also owned by the Physiological Society), Experimental Physiology.

Experimental Physiology is a peer-reviewed, Open Access journal covering all areas of physiology, with particular emphasis on integration and translation.

Further details can be found here: https://onlinelibrary.wiley.com/journal/10.1111/(ISSN)1469-445X.

You can find information on ways to pay Open Access Article Processing Charges (APCs), including a list of eligible consortia and institutions covered by Transformational Agreements with our publisher Wiley here: https://authorservices.wiley.com/author-resources/Journal-Authors/open-access/affiliation-policies-payments/institutional-funder-payments.html.

Since 2024, over 70% of manuscripts transferred to Experimental Physiology have gone on to receive an acceptance decision after review. Decisions for transferred manuscripts are also achieved 10 days faster on average than regular submissions to the journal.

We would strongly encourage you to address the review comments provided below. If you can demonstrate that you have done this (by providing a revised, highlighted version, plus a 'Response to JP Reviewers' file as part of your transferred submission), your manuscript is likely to undergo expedited review at Experimental Physiology by our Senior Editorial Team and not be sent out for de novo review by (another set of) specialists.

If you would like to proceed with transferring your manuscript for consideration by the Editorial Board of Experimental Physiology, please do so through the following link: Link Not Available.

If you have any questions about the transfer, or any specific reviewer comments, please don't hesitate to contact us. We will be happy to help.

Yours sincerely,

Vaughan Macefield
Senior Editor
The Journal of Physiology

EDITOR COMMENTS

Thank you for submitting your manuscript to The Journal of Physiology. We appreciate the time and care invested in this study.

I have carefully reviewed the expert referee's report and evaluated both their comments and your manuscript. The reviewer highlighted several strengths of your work, including the novelty of examining vascular absorption of venom-associated macromolecules, the in vivo physiological approach, and the interesting implications for understanding envenoming dynamics. At the same time, the reviewer identified a number of substantial concerns that would require considerable revision.

I hope that the feedback received here will help you further along with your manuscript.

Senior Editor:

Thank you for submitting your manuscript to The Journal of Physiology. I have now received comments from an independent reviewer and a reviewing editor, both experts in the field. While you present convincing physiology for Pseudonaja textilis venom, you do not provide viperid venom absorption data in your central FD-kinetics assay. The venoms were only tested in an extravasation context and not absorption. Addressing this gap would require new in vivo experiments and I do not believe that this could be delivered in a revision timeframe. Beyond new data, the reviewer identified multiple issues that would still need substantial correction. I am concerned that inviting a major revision will likely lead to multiple rounds of review, and possibly an eventual rejection, given that it does not dive deeply into the underlying physiology.

Nevertheless, given its scope, I believe your manuscript is suitable for consideration by our open-access sister journal, Experimental Physiology. If you wish to transfer to this journal we can arrange this seamlessly at our end.

REFEREE COMMENTS

Referee #1:

This study addressed a relevant topic which has received limited attention in snake venom research, i.e., the potential role of blood vasculature in the absorption of venom components and tissue products released in the course of envenoming. The authors demonstrate that fluorescent dextran particles are absorbed into the bloodstream after the injection of snake venom in the hind paw. Previous works had demonstrated the role of the lymphatic system in the systemic venom absorption from the tissue, and this work presents evidence indicating that blood vessels, mostly venules, also play a role in this absorption through the opening of paths in the vascular wall through which substances diffuse not only from the circulation to the tissue, but also from the tissue to the circulation. The study also demonstrates that the opening of these paths is associated with the action of inflammatory mediators derived from mast cells. Moreover, another valuable observation of the study is that, upon systemic absorption into the bloodstream, venom components are able to induce inflammation in distant tissues, facilitating the diffusion of venom components into the tissues. Overall, the study presents novel and relevant information that contributes to our understanding of the dynamics of venom absorption in envenoming. However, there are several issues that need to be improved for further considering this manuscript for publication.

(1) The title of the manuscript ("Bites from venomous snakes target an immune system response for optimal envenomation") does not fully represent the nature of the study. First of all, the word 'bites' does not correspond because this is an experimental study and bites would be related to actual envenomings. Then, it would be probably more precise to say that venoms elicit an inflammatory response for optimal envenomation instead of saying that targets an immune system response, which is a more comprehensive term of phenomena that were not investigated. I suggest modifying the title to better reflect what was done in the study, something like "Inflammation induced by snake venoms favors venom absorption through blood vessels and extravasation into tissues", or something like that.

(2) The authors mostly used one elapid venom in their experiments, and also included other elapid venoms and one viperid venom in some experiments. However, these additional venoms were used mostly in experiments that quantified the extravasation of albumin-Evans blue in the skin. It would have been interesting to assess the effect of Daboia venom in experiments measuring the absorption in the blood of fluorescent dextran upon hind paw injection. The reason for this suggestion is because viperid venoms not only promote inflammation and opening of pores in the vascular wall, but they also disrupt the integrity of blood vessels causing hemorrhage. What would be the role of such phenomenon in the absorption of fluorescent dextran into the blood? Would microvessel disruption by viperid venoms affect this route of venom absorption?

(3) Line 538 and thereafter: It is stated that "Our study demonstrates differences in the initiating mechanisms of elapid and viper venom-induced inflammation. The elapid venom (PtV) released permeability increasing factors by activating mast cells while the viper venom (DrV) utilised PIFs likely to be within the venom itself". I do not think the evidence presented allows such a generalization. The authors did not explore the absorption in the blood of dextran when using Daboia venom, thus it is not clear whether the disruptive action of this venom in the microvasculature might affect absorption into the blood. On the other hand, previous research by many groups have shown that inflammation induced by viperid venoms is also mediated through the generation of inflammatory mediators in the tissues. Furthermore, to do such generalization more viperid venoms should be used to assess whether there are differences in the effects induced. The rest of this paragraph is also highly speculative.

(4) The presence of markers in the blood may, at least in theory, result from the redistribution to the blood of components that were absorbed through the lymphatic vessels. How can this possibility be discarded based on the experimental protocol followed?

(5) In the experiments related to the extravasation of fluorescent dextran in the ear pinna, it is stated that " The ear pinna proved a representative site for imaging the tissue interstitium as ear clearance of FD10 consequent to IV injection of FD10 was some 10-fold slower (clearance half time (T0.5) = 56 min; 95% CI 29-237 min) than clearance in serum (T0.5 = 5.1 min; 282 95% CI 3.5-7.3 min) indicating that pinna imaging will reflect tissue dextran levels and not those in the blood (Fig. 2A)." However, a more convincing way to exclude the possibility that measurements also include dextran present inside blood vessels would be to perfuse saline solution through the vasculature in order to eliminate blood and thus leave only the dextran that is present in the tissue.

(6) Lines 90 and thereafter: the authors refer to NO, but there are many other inflammatory mediators that play a role, as demonstrated in the case of mast cells-derived mediators.

(7) Line 157: it is mentioned that the injection of venom was done in the hindpaw. Was it a subcutaneous injection? If so, please specify.

(8) Line 162: How was serum isolated? Please describe.

(9) The development of a mathematical model is described in the results section but is not mentioned in the materials and methods section.

(10) In my opinion there is too much text in the discussion on the therapeutic implications of these findings in terms of possible first aid interventions. Part of this discussion is speculative and not necessarily supported by the observations described in the study. It is suggested that the extent of the discussion on the practical application of these findings is reduced.

****

Response to Referee File

We sincerely appreciate the careful evaluation of this reasonably complex research and have incorporated key Referee suggestions in the revised manuscript. In addressing these concerns we declare that we have altered the title of the paper which now is: **"Inflammation induced by snake venoms optimises envenomation**". The primary changes are highlighted in sections of the "Manuscript with Changes" using blue font. We also note that we have re-analysed the measurement of the inflammatory activated pore (IAP) size, now averaging the plateau response with the 45 and 60 min time points and use MATLAB routines to provide pore size with 95% CIs (Methods Modelling").

Referee #1 wrote:

This study addressed a relevant topic which has received limited attention in snake venom research, i.e., the potential role of blood vasculature in the absorption of venom components and tissue products released in the course of envenoming. The authors demonstrate that fluorescent dextran particles are absorbed into the bloodstream after the injection of snake venom in the hind paw. Previous works had demonstrated the role of the lymphatic system in the systemic venom absorption from the tissue, and this work presents evidence indicating that blood vessels, mostly venules, also play a role in this absorption through the opening of paths in the vascular wall through which substances diffuse not only from the circulation to the tissue, but also from the tissue to the circulation. The study also demonstrates that the opening of these paths is associated with the action of inflammatory mediators derived from mast cells. Moreover, another valuable observation of the study is that, upon systemic absorption into the bloodstream, venom components are able to induce inflammation in distant tissues, facilitating the diffusion of venom components into the tissues. Overall, the study presents novel and relevant information that contributes to our understanding of the dynamics of venom absorption in envenoming. However, there are several issues that need to be improved for further considering this manuscript for publication.

(1) The title of the manuscript ("Bites from venomous snakes target an immune system response for optimal envenomation") does not fully represent the nature of the study. First of all, the word 'bites' does not correspond because this is an experimental study and bites would be related to actual envenomings. Then, it would be probably more precise to say that venoms elicit an inflammatory response for optimal envenomation instead of saying that targets an immune system response, which is a more comprehensive term of phenomena that were not investigated. I suggest modifying the title to better reflect what was done in the study, something like "Inflammation induced by snake venoms favors venom absorption through blood vessels and extravasation into tissues", or something like that.

We agree with this proposal and have modified the title, adopting a hybrid version of the two Referee suggestions. New title: "Inflammation induced by snake venoms optimises envenomation".

(2) The authors mostly used one elapid venom in their experiments, and also included other elapid venoms and one viperid venom in some experiments. However, these additional venoms were used mostly in experiments that quantified the extravasation of albumin-Evans blue in the skin. It would have been interesting to assess the effect of Daboia venom in experiments measuring the absorption in the blood of fluorescent dextran upon hind paw injection. The reason for this suggestion is because viperid venoms not only promote inflammation and opening of pores in the vascular wall, but they also disrupt the integrity of blood vessels causing

hemorrhage. What would be the role of such phenomenon in the absorption of fluorescent dextran into the blood? Would microvessel disruption by viperid venoms affect this route of venom absorption?

We compared several venoms including three other quite different elapid venoms and a viper venom to PtV action by measuring albumin exudation in response to subcutaneous venom injection. We agree that the viper venom DrV (Daboia russelii) being cytotoxic introduces the possibility that haemorrhage could confound interpretation. Transvascular pores large enough to pass red blood cells will have a radius of at least 250 nm (Deplaine *et al.*, 2011) and such pores if present will mediate substantial exudation of plasma. In contrast, venom absorption would be markedly inhibited due to the large outward flow of plasma through these large "pores". This is supported by our modelling which indicates that the relative absorption through haemorrhage-induced pores for vasculature near heart level will be 3% of that for IAPs (see Discussion "*The response to other venoms and the potential of haemorrhage being a confounding factor*" paragraph 1). Therefore, haemorrhage will not significantly contribute to direct venom absorption into the vasculature. In contrast, IAPs will continue to function normally despite the simultaneous presence of haemorrhage-associated pores. However, should haemorrhage lead to a reduction in venular pressure then the hydrostatic pressure range over which IAPs operate will be slightly increased.

Evidence against haemorrhage-associated pores subserving a substantial role in DrV-induced albumin exudation during the relatively short 1- 2 h time course of our experiments is provided by several findings. Haemorrhage onsets over much longer time-period than the acute vascular inflammatory response caused by opening of IAPs. The estimated haemorrhage onset time-period based on coagulopathy measured in humans for DrV is 0.5 – 12 h (Kularatne, 2003; Priyankara *et al.*, 2022), yet the DrV albumin exudation response commenced almost immediately (Fig. 3H). Importantly, we show that both PtV and DrV induce albumin exudation responses with profiles that are very similar and where a substantial component of the response is inflammation mediated given both responses were inhibited by ~ 50% by an antihistamine (Figs 3B & H). Furthermore, should haemorrhage induced pores have opened, then this would likely cause a substantial change in kinetics of the exudation response. This was not observed. Together, these findings are consistent with DrV-induced opening of IAPs and not haemorrhage-induced pores during our 1-2 h experiments (see Discussion section "*The response to other venoms and the potential of haemorrhage being a confounding factor*").

(3) Line 538 and thereafter: It is stated that "Our study demonstrates differences in the initiating mechanisms of elapid and viper venom-induced inflammation. The elapid venom (PtV) released permeability increasing factors by activating mast cells while the viper venom (DrV) utilised PIFs likely to be within the venom itself". I do not think the evidence presented allows such a generalization. The authors did not explore the absorption in the blood of dextran when using Daboia venom, thus it is not clear whether the disruptive action of this venom in the microvasculature might affect absorption into the blood. On the other hand, previous research by many groups have shown that inflammation induced by viperid venoms is also mediated through the generation of inflammatory mediators in the tissues. Furthermore, to do such generalization more viperid venoms should be used to assess whether there are differences in the effects induced. The rest of this paragraph is also highly speculative.

This was an observation that we found interesting. As considered in response to point 2 we find no evidence that Daboia venom had a disruptive action under the conditions of our experiment. We agree that "many groups have shown that inflammation induced by viperid venoms is also mediated through the generation of inflammatory mediators in the tissues" and note this in the Introduction. Our actions to these Referee comments have been to rename the header in the

associated Results section (now titled "*Exudation of albumin induced by several elapid venoms and a viper venom*") and to minimise speculation in the Discussion section (now titled "*Differences in initiation of elapid and viper venom-induced inflammation*").

(4) The presence of markers in the blood may, at least in theory, result from the redistribution to the blood of components that were absorbed through the lymphatic vessels. How can this possibility be discarded based on the experimental protocol followed?

In the Introduction we noted that published research indicates the lymphatics had a minor role in venom absorption at least initially for vasculature near heart level. As it transpires, we did investigate the lymphatic contribution to absorption of dextran FD10 in response to PtV finding that it had no significant effect and we now include this (see Results "*Inflammation-facilitated macromolecular absorption*"**,** paragraph 2). The mean fluorescence values normalised to control and measured ~ 2h after injection consequent to hind paw injection of PtV (0.1 mg/kg) + FD10 (1.5 mg/kg) + LGTN being 81 ± 27% (7) with LGTN compared to 100 ± 17% (8) for the PtV + FD10 control. This said, in the discussion we say more about the lymphatic contribution and note that even if small lymphatic uptake may affect measurements of absorption particularly for the largest dextran (see Discussion section titled "*Permeability of different sized macromolecules and the role of the lymphatics*").

(5) In the experiments related to the extravasation of fluorescent dextran in the ear pinna, it is stated that " The ear pinna proved a representative site for imaging the tissue interstitium as ear clearance of FD10 consequent to IV injection of FD10 was some 10-fold slower (clearance half time (T0.5) = 56 min; 95% CI 29-237 min) than clearance in serum (T0.5 = 5.1 min; 282 95% CI 3.5-7.3 min) indicating that pinna imaging will reflect tissue dextran levels and not those in the blood (Fig. 2A)." However, a more convincing way to exclude the possibility that measurements also include dextran present inside blood vessels would be to perfuse saline solution through the vasculature in order to eliminate blood and thus leave only the dextran that is present in the tissue.

We have re-investigated the clearance studies now using data where the infusion was done during the recording to obtain a true baseline. Having a defined baseline and using other data changed the decay time but it remained considerably longer than that for vascular clearance of FD10 (Fig. 2A). The Referee comment is quite useful as previously we had ignored the possibility of confounding fluorescence from the vasculature. As it transpires, our data allows additional analysis. The data indicates that there will be a contribution from the vasculature, but this is relatively small with the ear tissue providing ≥ 80% of the signal. This is presented in Results "*Inflammation facilitated macromolecular exudation from the bloodstream to body tissues*" paragraph 1).

(6) Lines 90 and thereafter: the authors refer to NO, but there are many other inflammatory mediators that play a role, as demonstrated in the case of mast cells-derived mediators.

We noted various other mediators in the earlier part of this Introductory paragraph and our use of histamine and serotonin antagonists confirms our interest in some of these PIFs. We presented an overview of NO as its vascular actions can vary and as it became integral in our experiments (see Fig. 2H).

(7) Line 157: it is mentioned that the injection of venom was done in the hindpaw. Was it a subcutaneous injection? If so, please specify.

Apologies for the oversight. It was subcutaneous (see Methods section "Procedures").

(8) Line 162: How was serum isolated? Please describe.

This methodology is now included in the Methods, *Procedures* (a)

(9) The development of a mathematical model is described in the results section but is not mentioned in the materials and methods section.

This is now included in the Methods section previously headed "Statistical analysis" which is now termed "*Statistical analysis and modell*ing".

(10) In my opinion there is too much text in the discussion on the therapeutic implications of these findings in terms of possible first aid interventions. Part of this discussion is speculative and not necessarily supported by the observations described in the study. It is suggested that the extent of the discussion on the practical application of these findings is reduced.

This Discussion section has been shortened and speculative discussion curtailed. We now use the Referee term "Therapeutic implications" as a header for this section in the Discussion. We have left in discussion relating to a methodology termed the "pressure pad technique" as this provides a way forward for the otherwise intractable problem of trying to stop small venoms from being absorbed using conventional PBI. We would like to present an anecdote that while not in manuscript provides some justification for its inclusion. It relates to an email from a Ranger in South Africa who informed us of his failure in using PBI against victims of MAMBA bites. He had no idea why it failed. He resorted to using a tourniquet and stated this was the only way he had some success. An explanation is that the active venom toxin in Mamba venom is dendrotoxin which is small and as such cannot be stopped by PBI as it needs a higher pressure than provided by conventional PBI. The use of tourniquets is of course extremely dangerous, but the suggested alternative pressure pad technique is not. It is for this reason that we elaborated on this. We don't consider it speculative as the methodology is published and is likely to be suitable for purpose though can remove this should it not meet approval.

References

Deplaine G, Safeukui I, Jeddi F, Lacoste F, Brousse V, Perrot S, Biligui S, Guillotte M, Guitton C, Dokmak S, Aussilhou B, Sauvanet A, Cazals Hatem D, Paye F, Thellier M, Mazier D, Milon G, Mohandas N, Mercereau-Puijalon O, David PH & Buffet PA. (2011). The sensing of poorly deformable red blood cells by the human spleen can be mimicked in vitro. *Blood* **117,** e88-95.

Kularatne SA. (2003). Epidemiology and clinical picture of the Russell's viper (Daboia russelii russelii) bite in Anuradhapura, Sri Lanka: a prospective study of 336 patients. *Southeast Asian J Trop Med Public Health* **34,** 855-862.

Priyankara S, Rathnasiri V, Mihiran T, Premawansa G, Isbister GK & Silva A. (2022). Mild venom-induced consumption coagulopathy associated with thrombotic microangiopathy following a juvenile Russell's viper (Daboia russelii) envenoming: A case report. *Toxicon* **212,** 8-10.

Dear Dr van Helden,

Re: JP-RP-2026-290494R1-A "**Inflammation induced by snake venoms optimises envenomation**" by Dirk Ferdinand van Helden, Neil James Spratt, Peter John Dosen, Sally Anne McFadden, John Holdsworth, Daniel J Beard, Kirsten Coupland, Phillip Jobling, Todd A Hardy, and Derek Laver

Thank you for submitting your manuscript to The Journal of Physiology. It has been assessed by a Reviewing Editor and by 1 expert referee and we are pleased to tell you that it is acceptable for publication following satisfactory revision.

The referee reports are copied at the end of this email.

Please address all the points raised and incorporate all requested revisions or explain in your Response to Referees why a change has not been made. We hope you will find the comments helpful and that you will be able to return your revised manuscript within 4 weeks. If you require longer than this, please contact journal staff: jp@physoc.org.

Your revised manuscript should be submitted online using the link in your Author Tasks Link Not Available. This link is accessible via your account as Corresponding Author; it is not available to your co-authors. If this presents a problem, please contact journal staff (jp@physoc.org). Image files from the previous version are retained on the system. Please ensure you replace or remove any files that are being revised.

If you do not wish to submit a revised version of your manuscript, you must inform our journal staff (jp@physoc.org) or reply to this email to request withdrawal. Please note that a manuscript must be formally withdrawn from the peer review process at one journal before it may be submitted to another journal.

TRANSPARENT PEER REVIEW POLICY: To improve the transparency of its peer review process The Journal of Physiology publishes online, as supporting information, the peer review history of all articles accepted for publication. Readers will have access to decision letters, including Editors' comments and referee reports, for each version of the manuscript, as well as any author responses to peer review comments. Referees can decide whether or not they wish to be named on the peer review history document.

ABSTRACT FIGURES: Authors are expected to use The Journal's premium BioRender account to create/redraw their Abstract Figures. Information on how to access this account is here: https://physoc.onlinelibrary.wiley.com/journal/14697793/biorender-access.

This will enable Authors to create and download high-resolution figures. If authors have used the free BioRender service, they can use the instructions provided in the link above to download a high-resolution version suitable for publication.

The link provided should only be used for the purposes of this submission. Authors will be charged for figures created on this account if they are not related to this manuscript submission.

LANGUAGE EDITING AND SUPPORT FOR PUBLICATION: If you would like help with English language editing, or other article preparation support, Wiley Editing Services offers expert help, including English Language Editing, as well as translation, manuscript formatting, and figure formatting at www.wileyauthors.com/eeo/preparation. You can also find resources for Preparing Your Article for general guidance about writing and preparing your manuscript at www.wileyauthors.com/eeo/prepresources.

RIGOUR AND REPRODUCIBILITY: We recommend authors consult The Journal's Rigour and Reproducibility requirements to ensure that their manuscript is suitable for publication: https://physoc.onlinelibrary.wiley.com/pb-assets/hub-assets/physoc/documents/TJP-Rigour-and-Reproducibility-Requirements.pdf

REVISION CHECKLIST:

Check that your Methods section conforms to journal policy: https://jp.msubmit.net/cgi-bin/main.plex?form_type=display_requirements#methods

Check that data presented conforms to the statistics policy: https://jp.msubmit.net/cgi-bin/main.plex?form_type=display_requirements#statistics

Upload a full Response to Referees file. To create your 'Response to Referees' copy all the reports, including any comments from the Senior and Reviewing Editors, into a Microsoft Word, or similar, file and respond to each point, using font or background colour to distinguish comments and responses and upload as the required file type.

Please upload two versions of your manuscript text: one with all relevant changes highlighted and one clean version with no changes tracked. The manuscript file should include all tables and figure legends, but each figure/graph should be uploaded as separate, high-resolution files. The journal is now integrated with Wiley's Image Checking service. For further details, see: https://www.wiley.com/en-us/network/publishing/research-publishing/trending-stories/upholding-image-integrity-wileys-

image-screening-service

You may also upload:

- 'Potential Cover Art' for consideration as the issue's cover image
- Appropriate Supporting Information (Video, audio or data set: see https://jp.msubmit.net/cgi-bin/main.plex?form_type=display_requirements#supp).

We look forward to receiving your revised submission.

If you have any queries, please reply to this email and we will be pleased to advise.

Yours sincerely,

Vaughan Macefield
Senior Editor
The Journal of Physiology

REQUIRED ITEMS

- You must start the Methods section with a paragraph headed Ethical approval (https://jp.msubmit.net/cgi-bin/main.plex?form_type=display_requirements#methods).

Research must comply with The Journal's policies regarding animal experiments (https://physoc.onlinelibrary.wiley.com/hub/animal-experiments) and adherence to these policies must be stated in the manuscript.

Authors should confirm in their Methods section that their experiments were carried out according to the guidelines laid down by their institution's animal welfare committee, including an ethics approval reference number. The Methods section must contain a statement about access to food, water and housing, details of the anaesthetic regime: anaesthetic used, dose and route of administration, and method of killing the experimental animals.

- Papers must comply with the Statistics Policy: https://jp.msubmit.net/cgi-bin/main.plex?form_type=display_requirements#statistics.

In summary:

- If n {less than or equal to}
30, all data points must be plotted in the figure in a way that reveals their range and distribution. A bar graph with data points overlaid, a box and whisker plot or a violin plot (preferably with data points included) are acceptable formats.

- If n > 30, then the entire raw dataset must be made available either as supporting information, or hosted on a not-for-profit repository, e.g. FigShare, with access details provided in the manuscript.

- 'n' clearly defined (e.g. x cells from y slices in z animals) in the Methods. Authors should be mindful of pseudoreplication.

- All relevant 'n' values must be clearly stated in the main text, figures and tables.

- The most appropriate summary statistic (e.g. mean or median and standard deviation) must be used. Standard Error of the Mean (SEM) alone is not permitted.

- Exact p values must be stated. Authors must not use 'greater than' or 'less than'. Exact p values must be stated to three significant figures even when 'no statistical significance' is claimed.

- Please include a full title page as part of your main article (Word) file, which should contain the following: title, authors, affiliations, corresponding author name and contact details, keywords, and running title.

EDITOR COMMENTS

Reviewing Editor:

Comments for Authors to ensure the paper complies with the Statistics Policy (Required):
Please ensure to report exact P-values in the manuscript text and in the graphs

Comments to the Author (Required):
Thank you for your careful revision.

The reviewer confirms their prior comments have been adequately addressed. The revision now clearly situates the finding within microvascular physiology, specifically inflammation-induced, pore-mediated macromolecular transport across venules and its consequences for macromolecular flux.

Before we proceed, I have a few housekeeping points:

1. As this study uses an animal model, please sure that the Methods include the standard ARRIVE-compliant information on animal housing and husbandry (including source and origin of animals and housing conditions and access to food and water). Please refer to the Information for Authors for details.

2. Please ensure that exact P-values are reported consistently in both the text and figure panels, in line with the Journal's statistical reporting guidance.

3. Minor typographical point: on line 697 there is a space in the unit for venular pressure ("mm Hg"), which should be formatted consistently.

4. Author profile: a profile photograph has been uploaded, but the accompanying textual author profile appears to be missing.

5. The abstract schematic explicitly notes use of ChatGPT to generate illustrative elements. Wiley's policy requires transparent disclosure of generative AI use beyond language editing, including for conceptual figures. Please include a brief disclosure statement (e.g. in the acknowledgements).

Please also see 'Required Items' above.

Senior Editor:

Comments for Authors to ensure the paper complies with the Statistics Policy (Required):
As per journal policy, please report exact p values unless $p<0.001$

Comments to the Author:
Thank you for submitting your fully revised manuscript to The Journal of Physiology. It has been assessed by the original reviewer and a reviewing editor and I am pleased to report that it will acceptable for publication following attendance to the small remaining matters.

REFEREE COMMENTS

Referee #1:

The authors have adequately addressed my comments, criticisms and suggestions to the first version of this manuscript.

END OF COMMENTS

JP RESPONSE 2026

Primary changes made are correction of minor improvements in our modelling including account for the reflection coefficient. These changes primarily affect scaling relating to prediction of pore size but do not impact on the experimental data or discussion points. We have also added figure 6F, a component to better interpret the consequences of venom-induced haemorrhage as presented in the Discussion (*The response to other venoms and the potential of haemorrhage being a confounding factor*)**.** These changes are marked in blue font.

We respond to Editor comments and journal protocol as follows:

J1) Please ensure you replace or remove any files that are being revised.

Revised Files replaced.

J2) ABSTRACT FIGURES: Authors are expected to use The Journal's premium BioRender account to create/redraw their Abstract Figures. Information on how to access this account is here: https://physoc.onlinelibrary.wiley.com/journal/14697793/biorender-access.

High resolution Abstract Figure made in part using Biorender has been submiited.

J3) RIGOUR AND REPRODUCIBILITY: We recommend authors consult The Journal's Rigour and Reproducibility requirements to ensure that their manuscript is suitable for publication: https://physoc.onlinelibrary.wiley.com/pb-assets/hub-assets/physoc/documents/TJP-Rigour-and-Reproducibility-Requirements.pdf

We have met Journal of Physiology "Rigour and Reproducibility" requirements.

J4) Methods section conforms to journal policy: https://jp.msubmit.net/cgi-bin/main.plex?form_type=display_requirements#methods

Conforms with journal policy

J5) Check that data presented conforms to the statistics policy: https://jp.msubmit.net/cgi-bin/main.plex?form_type=display_requirements#statistics

Data conforms to the Journal's statistics policy (see below)

J6) Please upload two versions of your manuscript text: one with all relevant changes highlighted and one clean version with no changes tracked. The manuscript file should include all tables and figure legends, but each figure/graph should be uploaded as separate,

high-resolution files. The journal is now integrated with Wiley's Image Checking service. For further details, see: https://www.wiley.com/en-us/network/publishing/research-publishing/trending-stories/upholding-image-integrity-wileys-image-screening-service

A clean version of our manuscript which includes figures and one with primary changes marked (blue font) have been included. Both have figures included (tables not used) and Modified figures have also been uploaded as separate, high-resolution png files.

REQUIRED ITEMS

J7) You must start the Methods section with a paragraph headed Ethical approval (https://jp.msubmit.net/cgi-bin/main.plex?form_type=display_requirements#methods).

Included.

J8) Research must comply with The Journal's policies regarding animal experiments (https://physoc.onlinelibrary.wiley.com/hub/animal-experiments) and adherence to these policies must be stated in the manuscript.
Included.

Authors should confirm in their Methods section that their experiments were carried out according to the guidelines laid down by their institution's animal welfare committee, including an ethics approval reference number. The Methods section must contain a statement about access to food, water and housing, details of the anaesthetic regime: anaesthetic used, dose and route of administration, and method of killing the experimental animals.

Included.

J9) Papers must comply with the Statistics Policy: https://jp.msubmit.net/cgi-bin/main.plex?form_type=display_requirements#statistics.
- If n {less than or equal to}
30, all data points must be plotted in the figure in a way that reveals their range and distribution. A bar graph with data points overlaid, a box and whisker plot or a violin plot (preferably with data points included) are acceptable formats.
- If n > 30, then the entire raw dataset must be made available either as supporting information, or hosted on a not-for-profit repository, e.g. FigShare, with access details provided in the manuscript.
- 'n' clearly defined (e.g. x cells from y slices in z animals) in the Methods. Authors should be mindful of pseudoreplication.
- All relevant 'n' values must be clearly stated in the main text, figures and tables.
- The most appropriate summary statistic (e.g. mean or median and standard deviation) must be used. Standard Error of the Mean (SEM) alone is not permitted.

Exact p values must be stated. Authors must not use 'greater than' or 'less than'. Exact p values must be stated to three significant figures even when 'no statistical significance' is claimed.

Data appropriately presented with *P* values or range of *P* values (for response-time plots)

J10) Please include a full title page as part of your main article (Word) file, which should contain the following: title, authors, affiliations, corresponding author name and contact details, keywords, and running title.

Included.

EDITOR COMMENTS

Reviewing Editor:

Comments for Authors to ensure the paper complies with the Statistics Policy (Required):
Please ensure to report exact P-values in the manuscript text and in the graphs

Exact *P* values now reported.

Comments to the Author (Required):
Thank you for your careful revision.

The reviewer confirms their prior comments have been adequately addressed. The revision now clearly situates the finding within microvascular physiology, specifically inflammation-induced, pore-mediated macromolecular transport across venules and its consequences for macromolecular flux.

Before we proceed, I have a few housekeeping points:

1. As this study uses an animal model, please sure that the Methods include the standard ARRIVE-compliant information on animal housing and husbandry (including source and origin of animals and housing conditions and access to food and water).

Please refer to the Information for Authors for details.

Jncluded (Methods, *Experimental model*)

2. Please ensure that exact P-values are reported consistently in both the text and figure panels, in line with the Journal's statistical reporting guidance.

P values now presented according to Journal guidelines.

3. Minor typographical point: on line 697 there is a space in the unit for venular pressure ("mm Hg"), which should be formatted consistently.

Corrected.

4. Author profile: a profile photograph has been uploaded, but the accompanying textual author profile appears to be missing.

Author profile now included on front page of manuscript

5. The abstract schematic explicitly notes use of ChatGPT to generate illustrative elements. Wiley's policy requires transparent disclosure of generative AI use beyond language editing, including for conceptual figures. Please include a brief disclosure statement (e.g. in the acknowledgements).

Included in acknowledgements.

Please also see 'Required Items' above.

Senior Editor:

Comments for Authors to ensure the paper complies with the Statistics Policy (Required):
As per journal policy, please report exact p values unless p<0.001

P values now conform to Journal guidelines.

Comments to the Author:
Thank you for submitting your fully revised manuscript to The Journal of Physiology. It has been assessed by the original reviewer and a reviewing editor and I am pleased to report that it will acceptable for publication following attendance to the small remaining matters.

REFEREE COMMENTS

Referee #1:
The authors have adequately addressed my comments, criticisms and suggestions to the first version of this manuscript.

Dear Professor van Helden,

Re: JP-RP-2026-290494R2 "**Inflammation induced by snake venoms optimises envenomation**" by Dirk Ferdinand van Helden, Neil James Spratt, Peter John Dosen, Sally Anne McFadden, John Holdsworth, Daniel J Beard, Kirsten Coupland, Phillip Jobling, Todd A Hardy, and Derek Laver

We are pleased to tell you that your paper has been accepted for publication in The Journal of Physiology.

The last Word (or similar) version of the manuscript provided will be used by the Production Editor to prepare your proof. When this is ready you will receive an email containing a link to Wiley's Online Proofing System. The proof should be thoroughly checked and corrected as promptly as possible.

Authors should note that it is too late at this point to offer corrections prior to proofreading. Major corrections at proof stage, such as changes to figures, will be referred to the Editors for approval before they can be incorporated. Only minor changes, such as to style and consistency, should be made at proof stage. Changes that need to be made after proof stage will usually require a formal correction notice.

All queries at proof stage should be sent to: TJP@wiley.com.

Following copyediting and typesetting the accepted version will be published online.

Yours sincerely,

Vaughan Macefield
Senior Editor
The Journal of Physiology

IMPORTANT POINTS TO NOTE FOLLOWING ACCEPTANCE OF YOUR PAPER:

• IMPORTANT NOTICE ABOUT OPEN ACCESS: To assist authors whose funding agencies mandate immediate public access to published research findings, The Journal of Physiology allows authors to pay an Open Access (OA) fee to have their papers made freely available immediately on publication.

The Corresponding Author will receive an email from Wiley with details on how to register or log in to Wiley Authors where you will be able to place an order.

• You can check if your funder or institution has a Wiley Open Access Account here:
https://authors.wiley.com/author-resources/Journal-Authors/open-access/author-compliance-tool.html

• TRANSPARENT PEER REVIEW POLICY: To improve the transparency of its peer review process, The Journal of Physiology publishes online as supporting information the peer review history of all articles accepted for publication. Readers will have access to decision letters, including Editors' comments and referee reports, for each version of the manuscript as well as any author responses to peer review comments. Referees can decide whether or not they wish to be named on the peer review history document.

• You can help your research get the attention it deserves! Check out Wiley's free Promotion Guide for best-practice recommendations for promoting your work at: www.wileyauthors.com/eeo/guide. You can learn more about Wiley Editing Services which offers professional video, design, and writing services to create shareable video abstracts, infographics, conference posters, lay summaries, and research news stories for your research at: www.wileyauthors.com/eeo/promotion.

• If you would like to receive our 'Research Roundup', a monthly newsletter highlighting the cutting-edge research published in The Physiological Society's family of journals (The Journal of Physiology, Experimental Physiology, Physiological Reports, The Journal of Nutritional Physiology and The Journal of Precision Medicine: Health and Disease), please click this link, fill in your name and email address and select 'Research Roundup':
https://www.physoc.org/journals-and-media/membernews

EDITOR COMMENTS

Reviewing Editor:

Thank you for your careful revision.

You have sufficiently addressed the comments we had in the last round, and I have nothing further to add.

Senior Editor:

Thank you for submitting your revised manuscript to The Journal of Physiology and for attending to the reviewer's comments. I am pleased to advise that the manuscript is now considered acceptable for publication.
